# Assessing walking ability using a robotic gait trainer: opportunities and limitations of assist-as-needed control in spinal cord injury

**DOI:** 10.1186/s12984-023-01226-4

**Published:** 2023-09-21

**Authors:** Serena Maggioni, Lars Lünenburger, Robert Riener, Armin Curt, Marc Bolliger, Alejandro Melendez-Calderon

**Affiliations:** 1 Hocoma AG, Volketswil, Switzerland; 2 ETH Transfer, ETH Zurich, Zurich, Switzerland; 3 Sensory-Motor Systems (SMS) Lab, ETH Zurich, Zurich, Switzerland; 4 Spinal Cord Injury Center, Balgrist University Hospital, Zurich, Switzerland; 5 School of Electrical Engineering and Computer Science, The University of Queensland, Brisbane, Australia; 6 School of Health and Rehabilitation Sciences, The University of Queensland, Brisbane, Australia; 7 Jamieson Trauma Institute, Metro North Health, Brisbane, Australia

**Keywords:** Assist-as-needed, Lokomat, Robotic gait training, Rehabilitation, Gait, Walking, Spinal cord injury, Assessment

## Abstract

**Background:**

Walking impairments are a common consequence of neurological disorders and are assessed with clinical scores that suffer from several limitations. Robot-assisted locomotor training is becoming an established clinical practice. Besides training, these devices could be used for assessing walking ability in a controlled environment. Here, we propose an adaptive assist-as-needed (AAN) control for a treadmill-based robotic exoskeleton, the Lokomat, that reduces the support of the device (body weight support and impedance of the robotic joints) based on the ability of the patient to follow a gait pattern displayed on screen. We hypothesize that the converged values of robotic support provide valid and reliable information about individuals’ walking ability.

**Methods:**

Fifteen participants with spinal cord injury and twelve controls used the AAN software in the Lokomat twice within a week and were assessed using clinical scores (10MWT, TUG). We used a regression method to identify the robotic measure that could provide the most relevant information about walking ability and determined the test–retest reliability. We also checked whether this result could be extrapolated to non-ambulatory and to unimpaired subjects.

**Results:**

The AAN controller could be used in patients with different injury severity levels. A linear model based on one variable (robotic knee stiffness at terminal swing) could explain 74% of the variance in the 10MWT and 61% in the TUG in ambulatory patients and showed good relative reliability but poor absolute reliability. Adding the variable ‘maximum hip flexor torque’ to the model increased the explained variance above 85%. This did not extend to non-ambulatory nor to able-bodied individuals, where variables related to stance phase and to push-off phase seem more relevant.

**Conclusions:**

The novel AAN software for the Lokomat can be used to quantify the support required by a patient while performing robotic gait training. The adaptive software might enable more challenging training conditions tuned to the ability of the individuals. While the current implementation is not ready for assessment in clinical practice, we could demonstrate that this approach is safe, and it could be integrated as assist-as-needed training, rather than as assessment.

**Trial registration:**

ClinicalTrials.gov Identifier: NCT02425332.

**Supplementary Information:**

The online version contains supplementary material available at 10.1186/s12984-023-01226-4.

## Background

Walking impairments affect around three quarters of stroke survivors [1] and the vast majority of people with a spinal cord injury (SCI) [2, 3]. Gait disorders are among the most frequent symptoms in neurology with more than 60% of neurological patients showing visible disturbances of gait [4]. In this work, we focus mainly on the SCI population. In 2016, there were 0.93 million new cases of SCI worldwide, and the prevalence of SCI patients currently living with the condition was 27.04 million [3]. More than half of SCI survivors present an incomplete lesion and have chances of recovering some walking function [2, 5]. Limitations in walking function affect independence, quality of life and lead to several secondary complications due to immobility, such as joint contractures, osteoporosis and spasticity [6, 7]. Walking recovery is among the highly desired goals of the rehabilitation for patients after stroke and spinal cord injury [6, 8, 9].

Patients with incomplete SCI who can walk present several kinematic abnormalities with respect to able-bodied control subjects, due to impaired proprioception, decreased voluntary muscular control, increased muscle tone and altered neural drive [10]. These individuals usually show slower walking speed and longer double support duration, limited hip and knee flexion during swing and insufficient hip extension during stance [10, 11].

Assessing and evaluating walking-related functions and activity is needed to monitor and adapt the therapy, to motivate patients and families and to provide evidence to health insurance companies. Research on new drugs and treatments requires sensitive assessments to capture the effects of therapeutic interventions. Nowadays, the assessment of walking and walking-related functions in clinical practice rely mainly on ordinal-based scores which suffer from several limitations, and there is no single outcome measure that can be used to monitor changes in lower extremity function regardless of severity and level of injury [12]. Time-based assessments, such as the 10-meter-walking-test (10MWT), provide useful information on overall performance, but they cannot capture the use of compensatory strategies, the quality of the gait pattern and they cannot be administered in people who have some residual function, but cannot walk yet. More sensitive assessments, such as camera-based gait analysis, require also more time for administering, thereby taking away time from therapy, and they are not often performed [13].

Since the early 2000s, robotic gait trainers have become valuable aids for the rehabilitation of walking after a neurological injury [14, 15]. Robotic gait trainers can provide intensive training with a high number of repetitions to patients with mild to severe gait impairments. Severely affected patients, who would not be able to train otherwise, can start earlier with the rehabilitation [16] thanks to the robotic support. Exoskeleton-type gait trainers (e.g. Lokomat [14], Walkbot [17]) control each of the leg segments (thigh and shank) independently and are programmed to generate a physiological hip and knee angular motion, i.e. similar to the gait pattern of an able-bodied individual. Because of the assistance that the robotic gait trainer provides, assessments can be administered even if the patient is not able to perform the movement without support.

The programmable logic of robotic devices can provide standard conditions for the assessments and the information from sensors can be used to calculate objective measures [18]. However, if the robot provides too much support, it is difficult to infer the capabilities of the patient and what he/she could do without the support of the device. On the other side, reducing the support to much would allow more deviation from the predefined gait pattern and could lead to potentially unsafe situations. For these reasons, manual tuning of guidance and support to be applied is challenging and subjective. To address this issue, we developed an adaptive controller in a treadmill-based robotic exoskeleton, the Lokomat [19]. The controller applies and assist-as-needed (AAN) logic [20]. The algorithm reduces the robotic support where possible, which encourages the patient to move actively, while maintaining a safe environment for training. Our hypothesis is that the converged level of robotic support (a combination of robotic joint impedance and body weight support) determined by the AAN algorithm is proportional to the patient’s level of walking impairment.

**Fig. 1:**
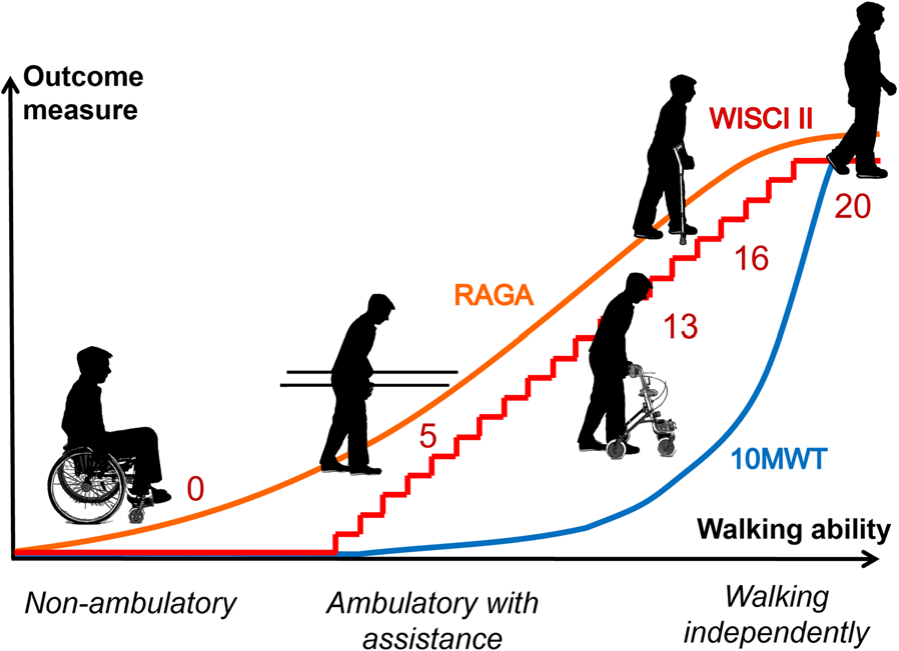
Robot-aided gait assessment (RAGA): existing outcome measures assessing gait score with “0” people that cannot yet walk, regardless of the underlying residual function (e.g. WISCI II, 10MWT). Moreover, many assessments are categorical (e.g. WISCI II). The long-term goal of this project is to develop an assessment of walking (Robot-aided Gait Assessment—RAGA) which can provide an objective, continuous score suitable to monitor the patient’s walking ability in all stages of rehabilitation. The Y axis shows a generic “outcome measure” value. The X axis represents patient’s walking ability, divided roughly in non-ambulatory, ambulatory with assistance and walking independently. Silhouettes are adapted with permission from [12]

Our work aims at developing a quantitative and objective evaluation of walking ability that (i) can be used during training and (ii) enables objective, valid, reliable and sensitive measurements. This should be applicable to patients with mild to severe gait impairments. Thus, even if an individual is not ambulatory, robotic devices could offer insights into walking-related functions, which is currently not possible with standard clinical assessments (Fig. 1).

The adaptive software was first tested in able-bodied subjects [19]. We also evaluated the system’s performance in assessing different types of typical walking impairments on a biomimetic robotic test bench [21]. This technical validation confirmed that the method can capture different levels of impairment and that the outcome measures are affected by speed. It also showed that the impairment is only visible in some gait phases.

In this paper, we tested the adaptive robotic controller in individuals with SCI and able-bodied subjects. Our aim was to study how the parameters of the controller adapted to the individuals’ motor impairment—from no impairment (able-bodied subjects) to severe walking impairment (individuals who were unable to walk)—and how they relate to ‘walking ability’ outside the robotic device. In our study, ‘walking ability’ is estimated with standard clinical scores related to walking speed (10MWT), speed and balance (timed-up-and-go—TUG) and isometric muscle force (L-FORCE). The subjects ‘walked’ in the Lokomat with the experimental AAN software. The robotic support values determined by the algorithm in several gait phases were used as measures to assess the individual’s walking ability.

In this exploratory study, we tested five hypotheses related to different aspects: (i) the assessment protocol is feasible in patients with different levels of gait impairment; (ii) the outcome measures captured by the AAN controller are able to provide relevant information about walking ability in ambulatory individuals with SCI; (iii) isometric muscle force measures could contribute to the prediction of walking ability; (iv) the Lokomat assessment is reliable; v) the extrapolation of these results applies also to non-ambulatory subjects and to unimpaired subjects.

## General methods

### Device and assist-as-needed controller

The robotic gait trainer used in this study was the Lokomat^®^Pro V5 (Hocoma AG, Switzerland). The Lokomat is a treadmill-based robotic exoskeleton with actuated hip and knee joints and a dynamic body weight support system that supports the patient through a harness. The orthosis is programmed to follow a predefined gait pattern with an impedance control strategy based on a reference trajectory. In the commercial version of the device, the gait pattern (reference trajectory), the mechanical impedance of the trajectory controller (known as ‘guidance force’) and the body weight support can be manually adjusted by the therapist through a user interface. High impedance values prevent pathological deviations from the reference hip and knee trajectories, but at the same time do not support the patient ‘as needed’ and do not allow therapists to observe active patient’s contribution to the movement.

To overcome these limitations, we modified the software of the commercial device and implemented an experimental adaptive controller [19, 22]. This adaptive (or “assist-as-needed” [20]) controller adjusts simultaneously (i) the mechanical impedance of the robot’s hip and knee joints throughout the different gait phases, based on the patient’s ability to follow a physiological gait pattern displayed on a screen (Fig. 3) and (ii) the body weight support (BWS).

In our study, the impedance of the joints starts from a default, high impedance in the machine (stiffness K_h_ = 1200 Nm/rad and damping B_h_ = 55 Nms/rad for the hip joint; stiffness K_k_ = 900 Nm/rad and damping B_k_ = 36 Nms/rad for the knee; this is equivalent to 100% GF). These robotic joint impedances are adapted independently at every step (at the end of each swing phase). The lower limit of the damping is coupled to the stiffness to guarantee stability. To optimally support each gait phase, 30 windows per step are implemented and the impedance is adapted separately in each window. The three windows during the stance phase are longer than the windows in the swing phase for stability reasons. For each window $w$ and for each step $s$ the joint impedance was defined by one set of parameters, ${\mathbf{K}}_{s,w}$ and ${\mathbf{B}}_{s,w}$**,** which was adapted according to the weighted kinematic error performed in each window and every step. Further details on the implementation of the controller and on the parameters used can be found in Additional file 3.

1 $${{\varvec{K}}}_{s+1,w}={\gamma }_{1}{\cdot {\varvec{K}}}_{s,w}+{g}_{1} \cdot {{f}_{1}\left({{\varvec{e}}}_{{\varvec{s}}}\right)}_{w}.$$

2 $${{\varvec{B}}}_{s+1,w}={\gamma }_{2}{\cdot {\varvec{B}}}_{s,w}+{g}_{2}{{\cdot f}_{2}\left({\dot{{\varvec{e}}}}_{{\varvec{s}}}\right)}_{w}.$$

A set of gains ${\gamma }_{1},{\gamma }_{2},{g}_{1},{g}_{2}$ were defined in order to have the impedance decrease slowly in the presence of physiological deviations and to react fast enough in case of large errors (Additional file 3). The impedance profile is smoothed before being applied to avoid abrupt changes in joint torques. The estimation of the subject’s performance is based on the kinematic deviation between the actual trajectory and the reference. However, individual trajectories can vary from the reference trajectory provided by the Lokomat, due to normal inter-subject variability [23, 24]. To cope with this problem, we implemented a mechanism to deal with physiological deviations that should not be considered as errors. Thresholds of maximum allowed deviations (lower $t{h}_{lo}$ and higher $t{h}_{hi}$) are determined around the reference angular trajectory ${{\varvec{q}}}_{ref}\left(i\right)$, where $i$ refers to a sample (typically at 1 kHz in the Lokomat). The thresholds are fixed for all subjects and are displayed in Additional file 3: Fig. A.3.1). The error weighting function ${{f}_{1}\left(\mathbf{e}\right)}_{s,w}$ consists of a hyperbolic tangent function of the kinematic error ${\varvec{e}}$ (Eq. 3) defined for each window $w$ and every gait step *s* (Eq. 4) [25]. The hyperbolic tangent function provides a smooth transition of the weighted error from 0 to 1 when ${{\varvec{q}}}_{act}\left(i\right)$ reaches the thresholds. The parameter *A* determines the slope of this transition. The function ${f}_{1}{\left({\varvec{e}}\right)}_{s,w}$ (Eq. 4) is then retained as error metric for the adaptation algorithm.

3 $${{\varvec{e}}}_{{\varvec{i}}}=\left({{\varvec{q}}}_{{\varvec{r}}{\varvec{e}}{\varvec{f}}}(i)-{{\varvec{q}}}_{{\varvec{a}}{\varvec{c}}{\varvec{t}}}(i)\right).$$

4 $${f}_{1}{\left({\varvec{e}}\right)}_{s,w}=\frac{1}{I}\cdot \sum_{i\in w}\left(1+\frac{1}{2}\cdot \left[tanh\left(A\cdot \left({{\varvec{e}}}_{{\varvec{i}}}-{\varvec{t}}{{\varvec{h}}}_{{\varvec{h}}{\varvec{i}}}\right)\right)-\mathrm{tanh}\left(A\cdot \left({{\varvec{e}}}_{{\varvec{i}}}+{\varvec{t}}{{\varvec{h}}}_{{\varvec{l}}{\varvec{o}}}\right)\right)\right]\right).$$

For each time point of the gait cycle, the subject’s hip and knee are allowed to deviate from the reference trajectory within the deadbands defined for each joint, independently from each other. Suitable deadbands in joint-space can be defined based on normal ranges for hip and knee joint angles (e.g. taking normative data from [24, 26, 27] or from able-bodied people walking in the device). In our case, we defined deadbands ad-hoc to ensure safety in critical phases of the gait cycle (e.g. terminal swing for correct foot placement) and at the same time allow physiological variability. The same approach is applied to deviations in velocity. The thresholds for lower and higher velocity error are symmetric with respect to 0.

The unloading of the body weight is adapted with a similar algorithm (Eq. 7). In this case the error metric is based on the difference $\Delta y$ between the actual ${(y}_{act})$ and the reference $({y}_{ref}$) heights of the hip center of rotation (CoR) during left and right single stance, similar to the approach presented in [28]. The height of the hip CoR (reference and actual) is estimated from the joint angles ${(q}_{hip}$: hip angle, ${q}_{knee}$: knee angle) and the subject’s segment lengths (Eq. 5, the same equation can be used to calculate ${y}_{act}$, using the actual angles). The rationale behind the choice of this metric is that the actual center of rotation of the hip will be lower than the reference height if the subject is not able to fully support his/her body weight during the stance phase.

5 $${y}_{ref}={l}_{1}\cdot \mathrm{cos}{(q}_{hip, ref})+{l}_{2}\cdot \mathrm{cos}{(q}_{knee, ref}-{q}_{hip, ref}).$$

6 $$\Delta {y}_{l}={y}_{ref,l}-{y}_{act,l.}$$

7 $${BWS}_{s+1,l}={\gamma }_{3}\cdot {BWS}_{s,l}+{g}_{3}\cdot {f}_{3}\left(\Delta {y}_{l}\right); l\in \left\{left,right\right\}.$$

8 $${f}_{3}\left(\Delta {y}_{l}\right)= \left\{\begin{array}{lll}\left(\Delta {y}_{l}-t{h}_{BWS}\right)\cdot BW & \quad if \Delta {y}_{l}\ge t{h}_{BWS} \\ 0 & \quad if \Delta {y}_{l}<t{h}_{BWS}.\end{array}\right.$$

9 $$t{h}_{BWS}=\left({l}_{1}+{l}_{2}\right)\cdot \left({{p}_{1}\cdot \left(1-\overline{K }\right)}^{4}+{p}_{2}\right).$$

A threshold $t{h}_{BWS}$ is determined taking into account the segment lengths and the mean stiffness K on the leg $l$ (Eq. 9). The threshold is higher for longer legs and lower stiffness values, since we expect higher displacements of the hip CoR in these conditions. If $\Delta {y}_{l}$ is higher than the threshold, the error is multiplied by the body weight *BW* of the patient, to ensure an increase in BWS proportional to the patient’s body weight. The values of the parameters used is reported in Additional file 3.

The initial BWS was set to 70% of the patient’s body weight, it could increase up to 80% and decreased until 5 kg.

**Fig. 2:**
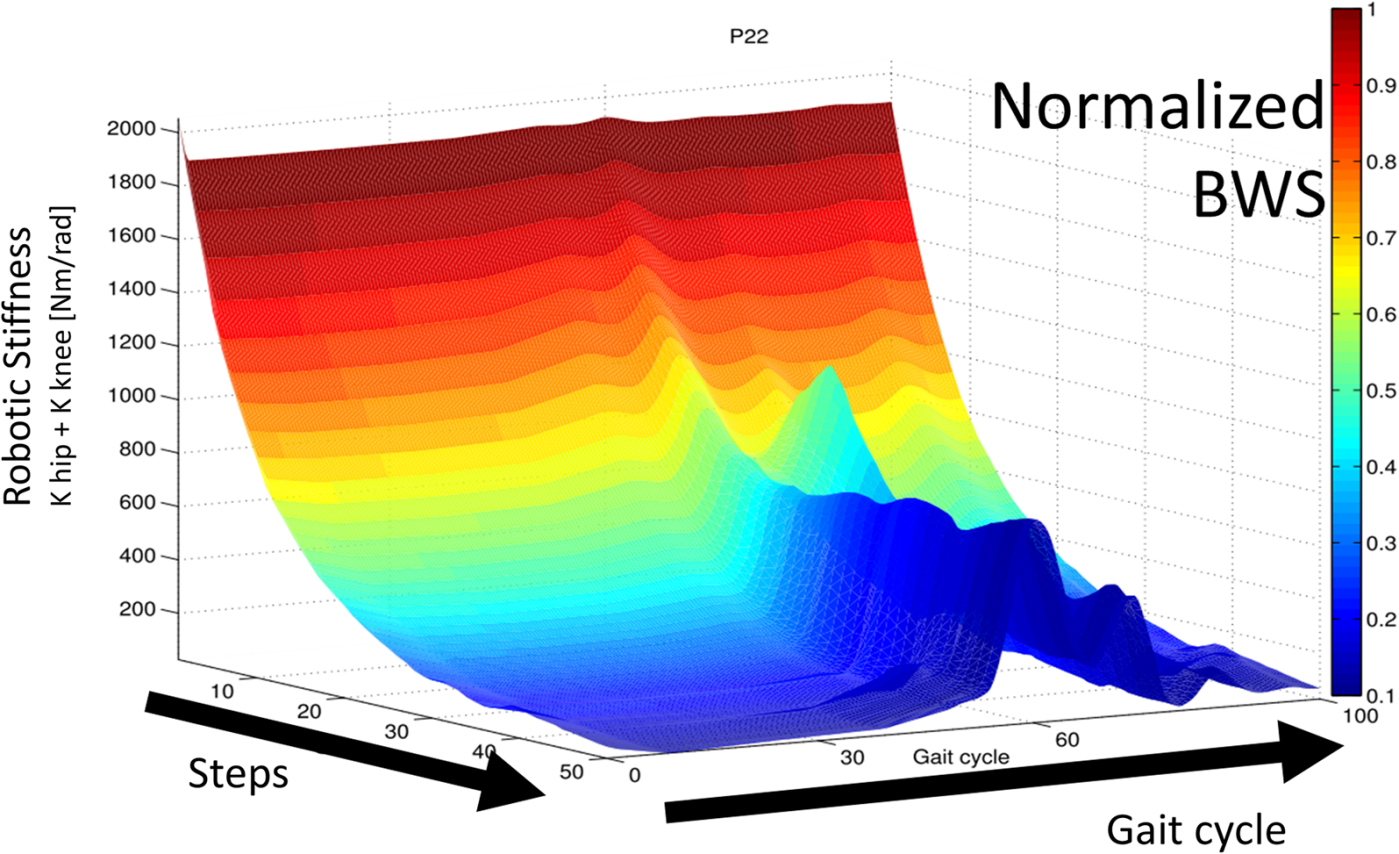
Adaptation of robotic support: robotic stiffness of hip and knee joint and normalized BWS during the adaptive task for subject P22. On the z axis, the sum of hip and knee stiffness is shown, while it decreases from step 1 to step 50 (y axis). For every step, the stiffness profile along the gait cycle is shown on the x axis. Stance phase lasts ca. from 0 to 60% of the whole gait cycle. The color is proportional to the BWS normalized by body weight

An example of the adapting impedance profile for a sample subject during 50 steps is shown in Fig. 2.

In this work, the adaptation was applied only at one leg at a time, while the other one was fully guided by the Lokomat. This choice was due to the fact that the visual feedback can only be provided on one leg at a time; moreover, we noticed in pilot tests that adapting the support of both legs at the same time is too physically demanding for patients [19].

### Population

The study was carried out at Balgrist Campus, Zurich, Switzerland between May and November 2016 and was approved by the Kantonale Ethikkommission Zürich (KEK-ZH-Nr. 2015-0020) and by Swissmedic (2014-MD-0035). A total of 27 subjects participated in this study: fifteen participants with a complete or incomplete SCI (age 54 ± 12, eleven males) and twelve unimpaired controls (age 43 ± 15, seven males) (Table 1). The inclusion criteria were: > 1 year post Spinal Cord Injury or persons without history of walking impairments. The exclusion criteria were: presence of contraindications to Lokomat training (a complete list can be found at https://www.hocoma.com/legal-notes/); inability or unwillingness to provide written informed consent or follow study procedures.

**Table 1:** Subject characteristics for the patient group (Pxx) and the able-bodied group (Sxx)

ID	Sex	Age [y]	Height [cm]	Weight [kg]	Time since lesion [y]	10MWT [m/s]	TUG [s]	BBS [1–56]	FAC [0–5]	AIS [A–D]	Level	WISCI II [0–20]	Notes/comorbidities
P22	M	57	172	72	5	1.89	4.80	56	5	D	T12	20	–
P24	M	49	178	78	9	1.69	5.15	56	5	D	C2	20	Neuropathic pain
P25	M	68	186	100	8	1.19	8.64	52	5	D	C2	20	Polytrauma with head contusion bleeding/mild cognitive deficit
P26	M	38	185	70	2	0.23	32.03	23	3	D	C7	16	–
P27	M	30	177	62	12	0.19	74	15	1	D	C6	11	Musculoskeletal and neuropathic pain
P37	M	53	179	100	7	0	NA	7	0	A	T11	8	Polytrauma
P40	M	54	180	87	11	1.78	6.88	56	5	D	C4	20	Chronic right lumboischialgia
P42	M	65	174	88	11	1.33	10.71	53	5	D	C2	20	Diabetic polyneuropathy
P43	F	51	165	78	< 1	1.27	8.63	56	5	D	T5	20	–
P44	M	56	178	80	ND	0	NA	6	0	D	C4	0	Multiple sclerosis
P45	F	74	163	73	13	0	NA	6	0	A	T10	0	–
P46	F	67	166	82	8	0.64	12.89	46	3	D	T4	16	–
P47	M	43	182	83	5	0.28	38.94	21	2	C	T12	12	–
P48	F	56	169	45	< 1	0.29	65.94	10	3	D	L1	13	–
P49	M	47	178	65	ND	0	NA	7	0	C	ND	0	–
S28	M	52	184	84		2.10	3.86	–	–	–	–	–	–
S29	M	29	188	77		2.14	5.77	–	–	–	–	–	–
S30	F	29	156	58		1.38	6.09	–	–	–	–	–	–
S31	F	25	160	55		1.97	5.30	–	–	–	–	–	–
S32	F	29	158	60		1.61	5.19	–	–	–	–	–	–
S33	M	53	170	65		2.59	4.44	–	–	–	–	–	–
S34	M	37	172	65		1.78	4.74	–	–	–	–	–	–
S35	F	61	164	60		2.34	3.78	–	–	–	–	–	–
S36	M	64	174	62		1.82	3.95	–	–	–	–	–	–
S38	M	45	171	58		2.01	4.06	–	–	–	–	–	–
S39	M	64	173	82		2.44	4.08	–	–	–	–	–	–
S41	F	26	166	58		2.61	4.91	–	–	–	–	–	–

NA: Not applicable (these patients could not perform the timed walking tests). ND: Not determined (missing data in the ASIA score sheet did not allow to determine the level of lesion precisely or date of lesion could not be retrieved)

### Experimental protocol

The study protocol consisted of three visits within 7 days (Table 2). During the first visit, a set of clinical assessments was carried out, followed by a familiarization phase with the AAN algorithm. The assessments focused on: gait speed, balance, assistance required for walking, muscle force, functional status (Table 3).

**Table 2:** Experimental protocol: assessments performed on each visit

Visit 1	Visit 2	Visit 3
Clinical assessments L-FORCE Familiarization	AAN-based assessment TLX Questionnaire	AAN-based assessment TLX Questionnaire

**Table 3:** Other clinical assessments included in the experimental protocol

Domain	Test	Abbreviation and reference
Gait speed	10 meter walking test (fastest speed) Time up-and-go test	10MWT [29] TUG [29]
Balance	Berg Balance Scale Time up-and-go test	BBS [30] TUG [29]
Muscle strength	L-FORCE—Lokomat isometric joint torque assessment (hip/knee, flexion/extension) Manual muscle test	LF [31] MMT [32]
Assistance required for walking	Walking index for spinal cord injury Functional ambulation category	WISCI II [29] FAC [30]
Functional status	ASIA impairment scale	AIS [33]

All the assessments were performed during the first visit. We recorded the fastest walking speed during the 10MWT, since we think that fastest walking speed would reflect better patients’ walking capacity (what an individual can do in a ‘standardised’ environment—in the ICF terminology) and their potential to participate in a community challenge [2]

**Fig. 3:**
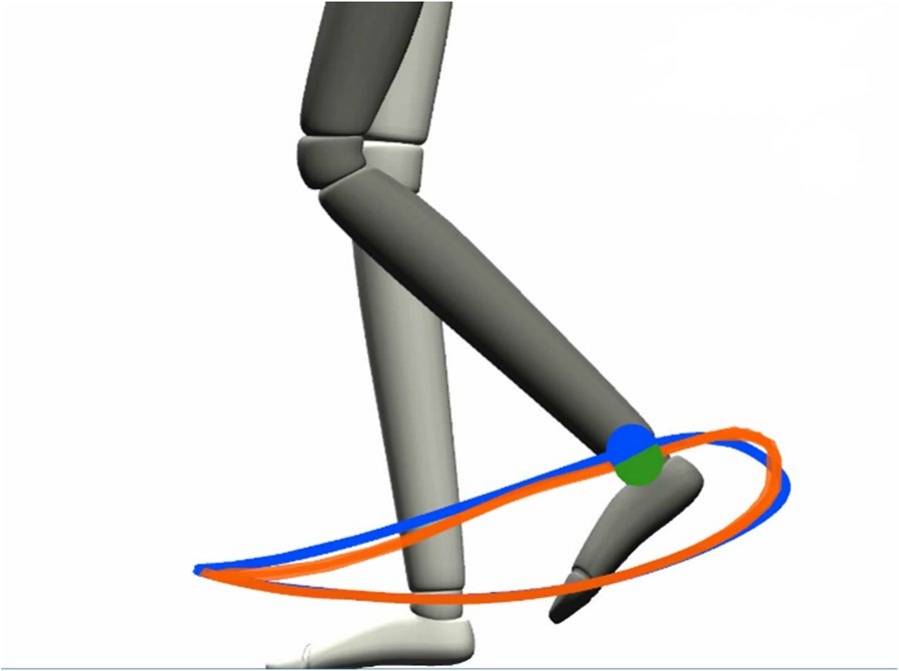
Visual feedback provided to the subjects. The reference trajectory and reference position are shown in blue, while the actual trajectory is shown in orange. The actual position is indicated by a green dot when the error is in a physiological range, changing to red when the error is outside the deadbands defined in the adaptive controller

In the able-bodied (AB) control group, only the 10MWT, TUG and L-FORCE were conducted. During the Lokomat familiarization phase, the subjects were set up in the Lokomat for a practice session. The settings were adjusted until the most suitable gait pattern for the subject was found, based on the manufacturer guidelines.^1^ Moreover, we used the patient’s actual foot trajectory and the robot-imposed trajectory displayed on the screen (Fig. 3) as guidance, trying to match them as much as possible (the reference trajectories applied in the study for all participants are displayed in Additional file 3: Fig. A.3.2). The Lokomat settings obtained in the first session (cuff size, leg length, hip and knee offset and range of motion) were retained and used on the next two sessions. An isometric joint torque assessment (L-FORCE) [31] was performed in the Lokomat before the subjects started to walk. L-FORCE assesses the isometric torque generated by the patient in a static position for flexor and extensor muscle groups in the hip and knee joints. The patient is lifted completely above the treadmill (no foot contact) and the Lokomat moves the patient’s legs in a predefined position (30° hip flexion and 45° knee flexion). The therapist can then sequentially start the measurement for each joint and each direction (e.g. knee flexion, knee extension and so on). The patient is asked to exert the maximum joint torque during the assessment, which is executed once per joint and direction. The torque generated by the patient is displayed in real-time on the screen. The subjects walked in the Lokomat with a speed of 1.9 km/h with full robotic support (40 steps) and then using the adaptive controller (50 steps) (Table 4). The subjects received instructions on the task: they were requested to follow the blue trajectory in space and in time (the blue dot indicated the desired position at every instant) (Fig. 3). Subjects were not permitted to use the handrails. The adaptive task was limited to one leg at a time, to ensure a proper display of the reference and actual trajectory and reduce the cognitive and motor demand on the patients performing the task. The foot trajectory performed in the last step was shown in orange. After each run with the adaptive controller, the Lokomat support was set back to the initial conditions (100% guidance force (GF) and 70% BWS).

**Table 4:** Experimental protocol sequence

# Steps	40	50	40	50
Task	Max support (100% GF and 70% BWS)	Adaptive impedance leg 1 + Adaptive BWS	Max support (100% GF and 70% BWS)	Adaptive impedance leg 2 + Adaptive BWS

*GF* guidance force, *BWS* body weight support

During the second and third visit the adaptive algorithm was used and data was recorded. Next, the adaptive software (all tasks in Table 4) was executed first at 1.6, then at 1.9 and finally at 2.2 km/h. Only the middle speed was analyzed in this study. Left leg and right leg were tested separately, according to the sequence shown in Table 4. The leg tested first in every subject was randomly chosen between left and right.

At the end of each visit, the subjects were asked to fill out the NASA Task Load Index (TLX) questionnaire, which is a subjective assessment tool that rates perceived workload in performing a task that foresees a human–machine interaction. It rates performance across six dimensions (mental, physical and temporal demand, perceived effort, performance and frustration), to determine an overall workload rating [34].

### Data preparation

**Fig. 4:**
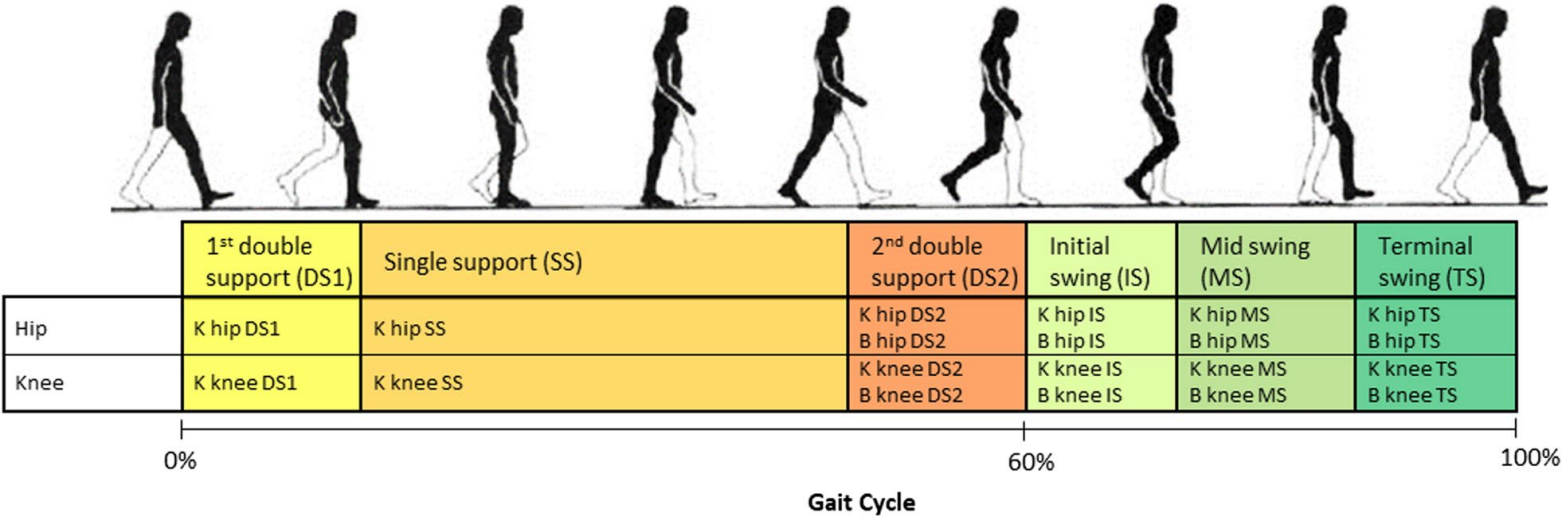
Strides were divided in 6 gait phases: first double support (DS1), single stance (SS), second double support (DS2), initial swing (IS), mid swing (MS) and terminal swing (TS). For every gait phase the stiffness K and the damping B (except in DS1 and SS) are calculated as the average between left and right leg

Data analyses and statistics were done with Matlab R2016b. Steps were divided and segmented in 6 gait phases (Fig. 4) using the heel strike and toe-off instants as described in [35]. The swing phase was divided in three sub-phases according to gait literature [36].

The impedance of the robotic joints and the BWS decrease following an exponential decay, until a point where the error prevents a further decrease. For each gait phase, the robotic impedance of hip and knee joint (K hip, K knee, B hip, B knee) was averaged and modeled as exponential decay to filter out inter-step noise. The average over the last 5 modeled steps of each adaptive task (left and right leg adaptation) was calculated, and afterwards averaged over the left and right task. The modeled BWS averaged on the last 5 steps of each adaptive task was also calculated and normalized by the individual’s body weight. The residual impedance was used as an indicator of the support required for walking in the different gait phases. The stiffness K was adapted based on spatial deviation from the predefined trajectory. Thus, K reflects the additional support required to follow the reference trajectory in space. The damping B was adapted based on errors in velocity and reflects, therefore, the additional support required to follow the trajectory with an adequate speed. We did not consider the damping B in the first double support phase (DS1) and single stance (SS) since the speed error is less relevant for these phases (the speed of the treadmill determines the speed of the foot). The residual BWS indicates the support required during stance phase. The BWS was then averaged among left and right task. For each subject, a dataset for the second visit and a dataset for the third visit were considered.

The L-FORCE results for each joint and each direction were averaged over left and right leg and normalized by body weight. For each joint and direction, the maximum value among all the subjects was found and used to normalize all the other subjects’ values.

### Presentation of results

Given the exploratory nature of this first study in individual with SCI, and to improve readability, the remaining of the paper is organized in sections, each reporting a question of interest, methodologies and results.

## Is the AAN controller feasible for Individuals with SCI?

Our first aim of this study was to evaluate the feasibility, and the mental and physical demand of the assessment protocol. To evaluate these aspects, we conducted a NASA-TLX questionnaire. The correlation between the NASA-TLX results and the clinical scores was studied using the Spearman correlation coefficient.

### Results

**Fig. 5:**
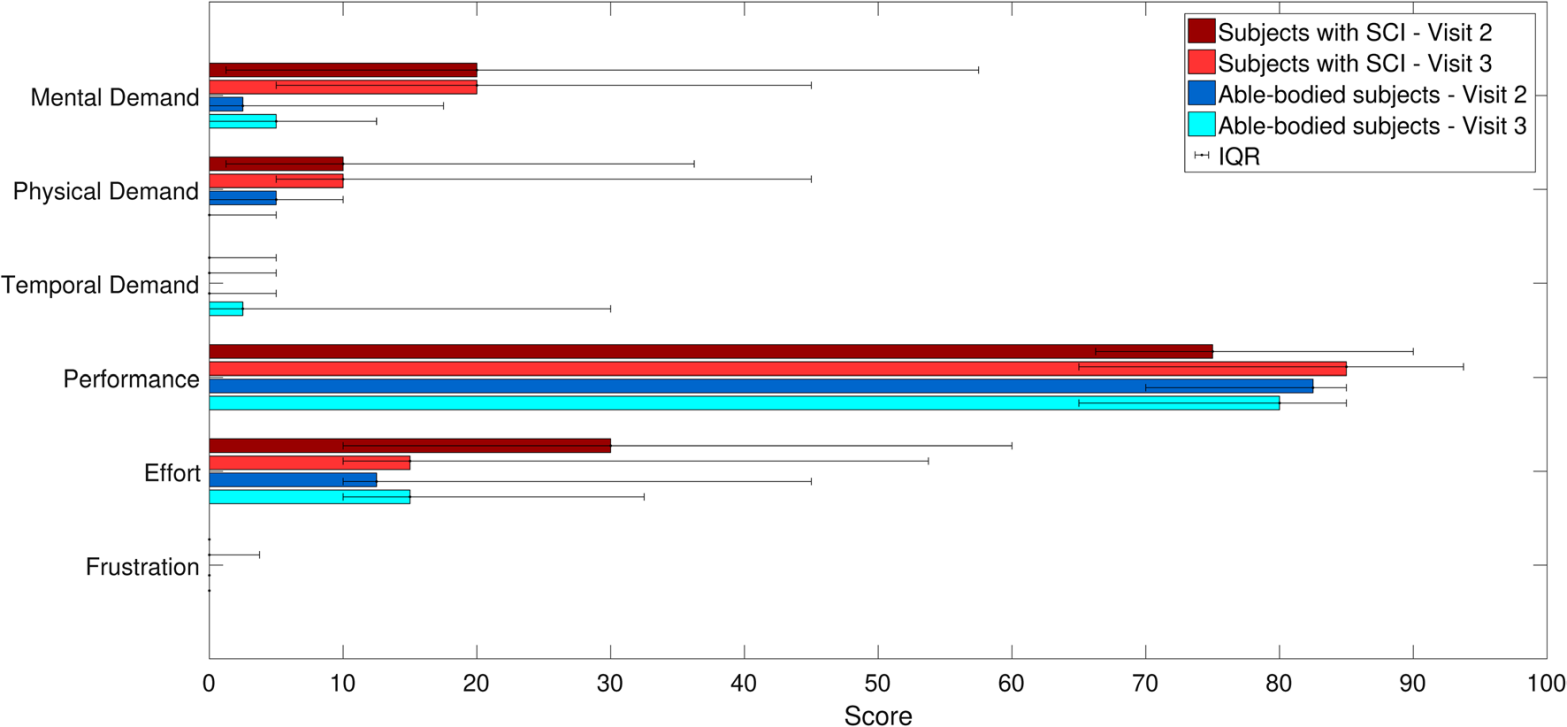
Mean and interquartile range of the answers to the NASA TLX questionnaire, grouped by visit and participant group

All subjects (both individuals with complete and incomplete SCI) were able to perform the assessment protocol with the AAN controller, without the occurrence of any adverse event. The NASA-TLX questionnaire (Fig. 5) showed perceived mean mental and physical demand below 25% both for patients and able-bodied subjects. Patients rated the task more mentally rather than physically demanding. In ambulatory patients, the perceived physical demand correlated strongly with the clinical scores (10MWT: ρ = − 0.93, TUG: ρ = − 0.80; WISCI: ρ = − 0.79) (see Additional file 2). Performance was rated as high (above 75%) by all subjects. Effort was rated around 30% of the maximum scale by patients during the first session and it decreased of a half during the second session. Frustration was not reported in either population.

## What are the most representative robotic variables that explain walking ability?

Our second aim was to identify the most representative variables determined by the AAN controller in the Lokomat that relate to ‘walking ability’ (as measured by the 10MWT and TUG) in individuals with SCI. Linear regression was used as an exploratory technique to identify the best predictor(s) of walking speed, rather than to determine an accurate prediction model [37]. Only ambulatory patients, i.e. patients that had a speed higher than 0 m/s in the 10MWT, were included in this analysis (*n* = 11).

We included all the variables listed in Fig. 4 and the BWS and we used Lasso (Least Absolute Shrinkage and Selection Operator) as a variable selection algorithm [38] (details can be found in Table 5). Lasso is a least-square regression method that adds a penalty term equal to the sum of the absolute values of the coefficients, multiplied by a parameter δ. The addition of the penalty term forces the coefficients of the linear model to shrink and sets some coefficients to 0. We used the function *lasso* in Matlab R2016b with a fivefold cross-validation to determine the parameter δ.

**Table 5:** Steps for the selection of predictor(s) and evaluation of the model

**Steps for selecting the predictor(s)** 1. Run Lasso on 1000 bootstrap samples (Bolasso) 1.1. Select *n-3* subjects without replacement at every run 1.2. For every subject, select randomly either observation from visit 2 or observation from visit 3 1.3. Run Lasso on the selected sample and save the vector of coefficients β 2. Select all the variables with coefficient ≠ 0 in ≥ 60% of the cases **Steps for bootstrap evaluation of the model** 3. Run *fitlm* on 1000 bootstrap samples 3.1. Select *n-3* subjects without replacement at every run 3.2. For every subject, select randomly either observation from visit 2 or observation from visit 3 3.3. Run *fitlm* on the selected sample and save β, Adj. R^2^ and residuals 4. Calculate the average model coefficients β and their CI, the CI of the mean and the PI for new observations	

*β* vector of model coefficients, *CI* confidence interval, *PI* prediction interval

We included two observations per patient (data from visit 2 and visit 3) and we implemented a bootstrapping procedure to study if the predictors selected via Lasso were consistent (Bolasso) [39, 40]. We reported the results from 1000 bootstrap replications in the Additional file 1. At every replication, we selected at random *n − 3* subjects without replacement, choosing for each randomly selected subject at every run either the observation from visit 2 or the observation from visit 3. By selecting for each subject only one observation, we tried to limit the effect of having two dependent observations in the same pool. We obtained 1000 vectors of coefficients. We selected only the predictor(s) whose coefficient was different from 0 in at least 60% of the replications.

We then generated unregularized linear models using the *fitlm* function within a second bootstrap loop to determine the adjusted R^2^, the Confidence Interval (CI) of the coefficients and the regression line and the Prediction Interval (PI) for new observations. At every bootstrap replication, *n − 3* subjects were selected without replacement as explained above, including randomly either the observation from visit 2 or from visit 3. We reported the values of the coefficients of the model to give an idea of the association between the selected predictor(s) and the predicted variables.

We repeated the same procedure described above for predicting the TUG. The data from the TUG were reciprocated, to have them in a similar form to the data of the 10MWT, which are expressed as speed (m/s). This allows the TUG data of patients who could not perform the test to be expressed as numeric value (0 s^−1^).

### Results

#### Robotic variables that explain walking ability as measured by 10MWT

**Fig. 6:**
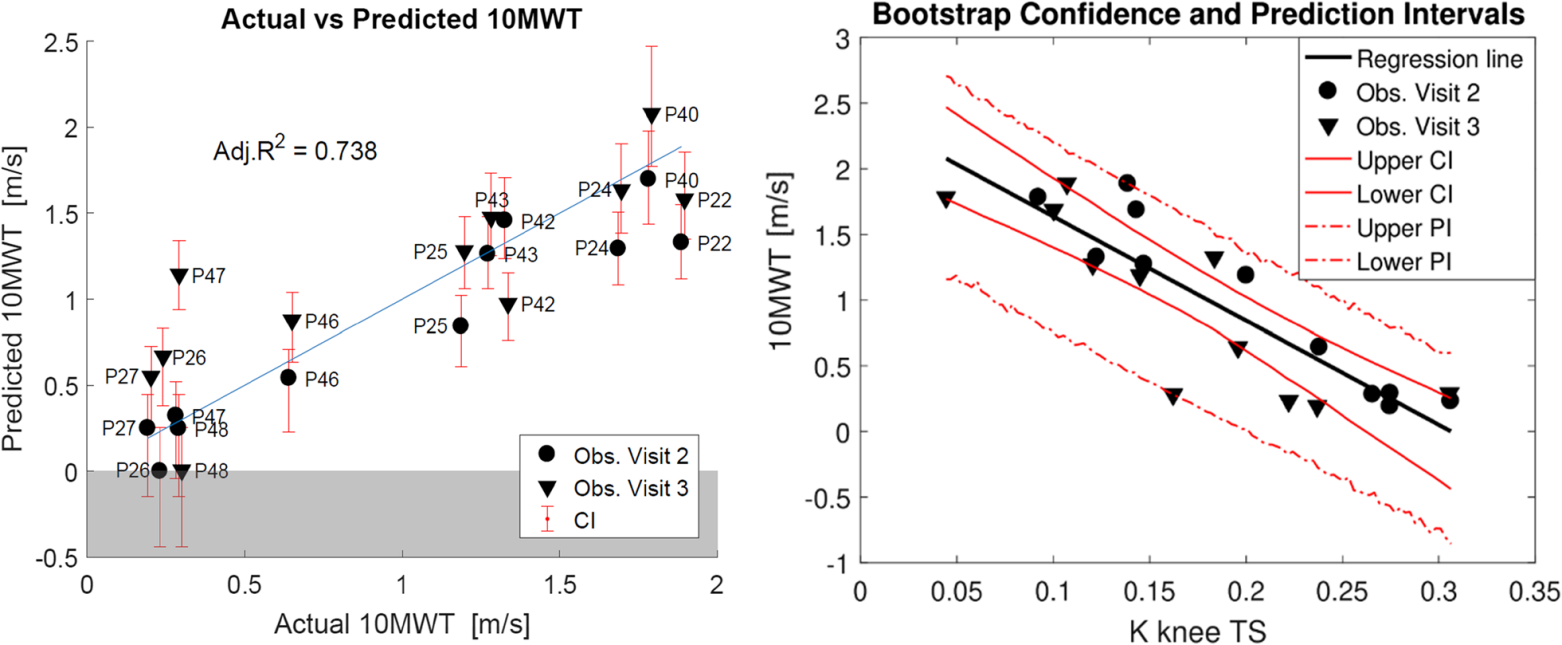
Left: actual vs predicted 10MWT: the unregularized linear model is created with the variable selected with Bolasso, K knee TS. The error bars show the confidence interval of the predicted observations. In blue, the identity line is shown. Right: The predictor K knee TS vs the predicted 10MWT (black line). CI (continuous red line) and PI (dashed red line) are calculated from the second round of bootstrapping

In 1000 bootstrap replications of Lasso, the variable K knee TS (knee stiffness at terminal swing) was selected 87.3% of the time (see Additional file 1: Fig. A.1.1 for percentage of selection of all the variables). We used only this predictor to generate the unregularized linear model to predict the 10MWT in ambulatory patients, as no other variable was selected in more than 60% of the replications. K knee TS was a significant predictor of speed in the 10MWT (β = − 7.933, Confidence Interval (CI) = [− 11.072, − 6.189], p < 0.001). The coefficient in the model was negative, meaning that the higher the support required from the knee at terminal swing, the lower was the speed measured in the 10MWT. The adjusted R^2^ for the model generated from all the observations was 0.738 (CI = [0.420, 0.936]) and the average Prediction Interval (PI) was 1.410 m/s (Fig. 6).

#### *R*obotic* v*ariables that* e*xplain* w*alking* ability* as* measured by TUG*

**Fig. 7:**
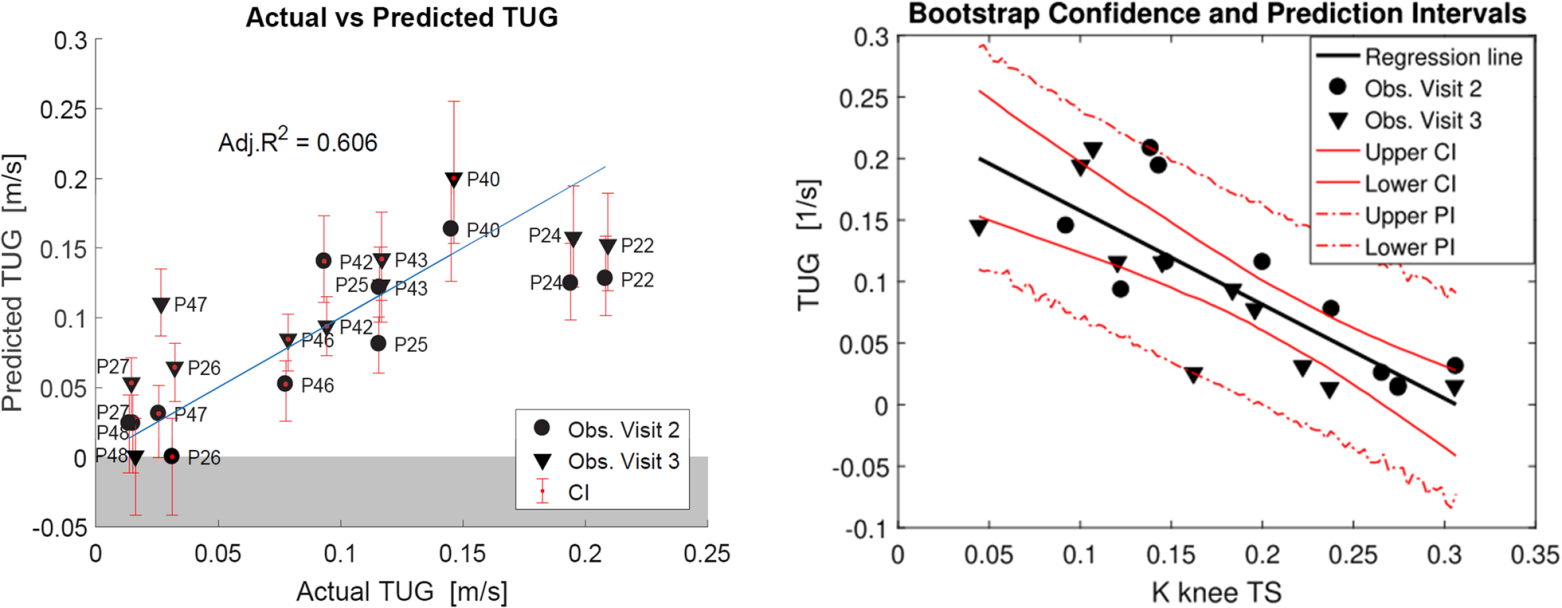
Left: actual vs predicted TUG: the unregularized linear model is created with the variable selected with Bolasso, K knee TS. The error bars show the confidence interval of the predicted observations. In blue, the identity line is shown. Right: the predictor K knee TS vs the predicted TUG (black line). CI (continuous red line) and PI (dashed red line) are calculated from the second round of bootstrapping. The reciprocal of the TUG data in seconds are used for the model, to make it consistent with the 10MWT data, which are expressed in m/s

During bootstrapping for the prediction of TUG, K knee TS was again selected most frequently, but less often than for the prediction of the 10MWT (75.9% of the times in 1000 bootstrap runs—see Additional file 1: Fig: A.1.2). The coefficient of K knee TS was negative, as for the 10MWT (− 0.763, CI: [− 1.098, − 0.517]). In the unregularized model, K knee TS was a significant predictor (p < 0.001), with an adjusted R^2^ of 0.606 (CI = [0.420, 0.936]) and an average PI of 0.166 s^−1^ (Fig. 7).

## Do force measures contribute to the prediction of walking ability?

Our third aim is to check if muscle strength contributes to the prediction of walking ability (as measured by the 10MWT and TUG). Muscle strength of the lower limbs is highly related to walking speed [41, 42]. Therefore, we investigated if measures of isometric forces (L-FORCE) improve the prediction of walking speed as measured in the 10MWT and TUG and if they are better predictors than the AAN outcome measures, i.e. will L-FORCE measures be chosen in the Bolasso procedure together or even instead of the AAN outcome measures?

To check this, we added the maximum voluntary isometric torque values (Hip Flexion (LF_HF), Hip Extension (LF_HE), Knee Flexion (LF_KF), Knee Extension (LF_KE)) to the dataset described in the previous section. For every joint and direction, we took the average torque among left and right leg and we z-normalized each variable. We then applied the same method described in Table 5 to select the best predictor(s) of walking speed among the AAN outcome measures and the isometric force data. Also in this case, only ambulatory patients were included. The same procedure was applied to predict the TUG. The correlations between the different L-FORCE measures were studied using the Spearman correlation coefficient.

### Results

#### Force measures that explain walking ability as measured by 10MWT

After adding the maximum voluntary isometric torque values to the AAN data, in 1000 bootstrap replications of Lasso, the variable K knee TS was again selected in most of the cases (81.4%—see Additional file 1: Fig. A.1.3). The second most selected variable was hip force LF_HF (66.1%). When both variables were used to generate an unregularized linear model to predict the 10MWT, the Adjusted R^2^ was 0.857 (CI = [0.661, 0.980]).

**Fig. 8:**
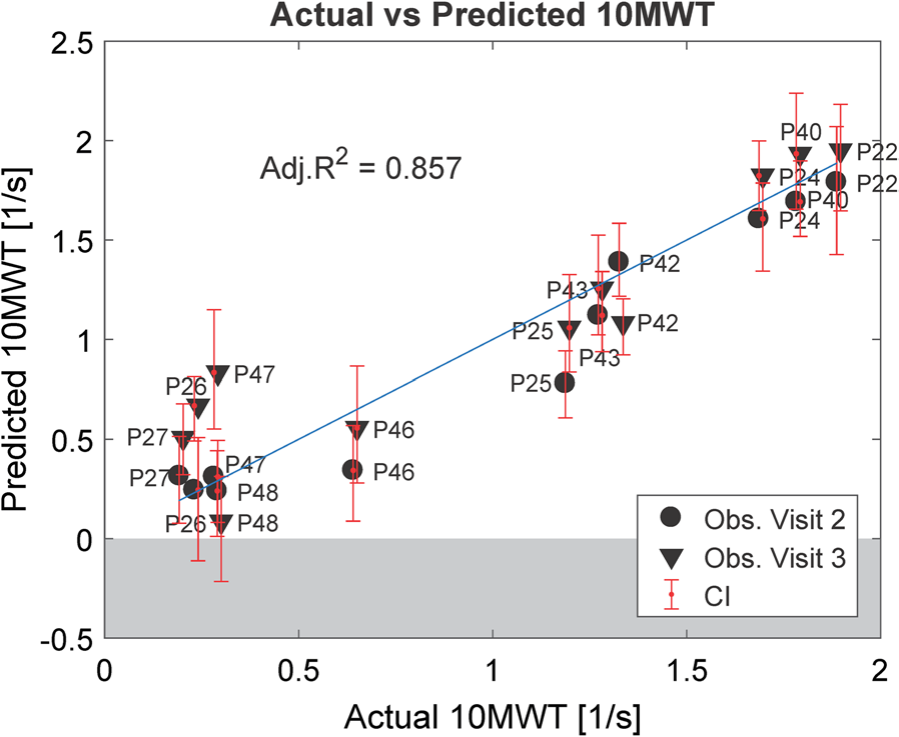
Prediction of 10MWT based on force and AAN data: Unregularized linear model for predicting the 10MWT with 2 predictors (K knee TS and LF HF). In blue, the identity line is shown

The coefficient of LF_HF was positive (β = 1.405, CI = [0.336, 2.295], p < 0.001), since, as expected, higher isometric force at the hip leads to higher speed in the 10MWT. The average PI decreased to 1.147 m/s (− 0.263 m/s) compared to the model with a single predictor (Fig. 8).

#### Force measures that explain walking ability as measured by TUG

In the prediction of the TUG, instead, LF_KF was selected 75.9% of the times in 1000 bootstrap replications, while K knee at terminal swing was only selected 62.8% of the times (see Additional file 1: Fig. A.1.4). When looking at the percentage of selection of the other L-FORCE measures, we found that LF_HF was selected 50% of the times. Maximum knee flexion torque was highly correlated with maximum hip flexion (ρ = 0.92). It may be, therefore, that this other L-FORCE variable could be used to predict the TUG with a similar accuracy. While Lasso is suggested as technique to handle datasets with multicollinearity [43], it cannot completely solve this issue. We removed LF_KF from the analysis and we ran again the Bolasso procedure to check if other force measures were picked over the AAN outcome measures: the hip flexion LF_HF was selected 82.9% of the times, while K knee TS only 60.7%.

**Fig. 9:**
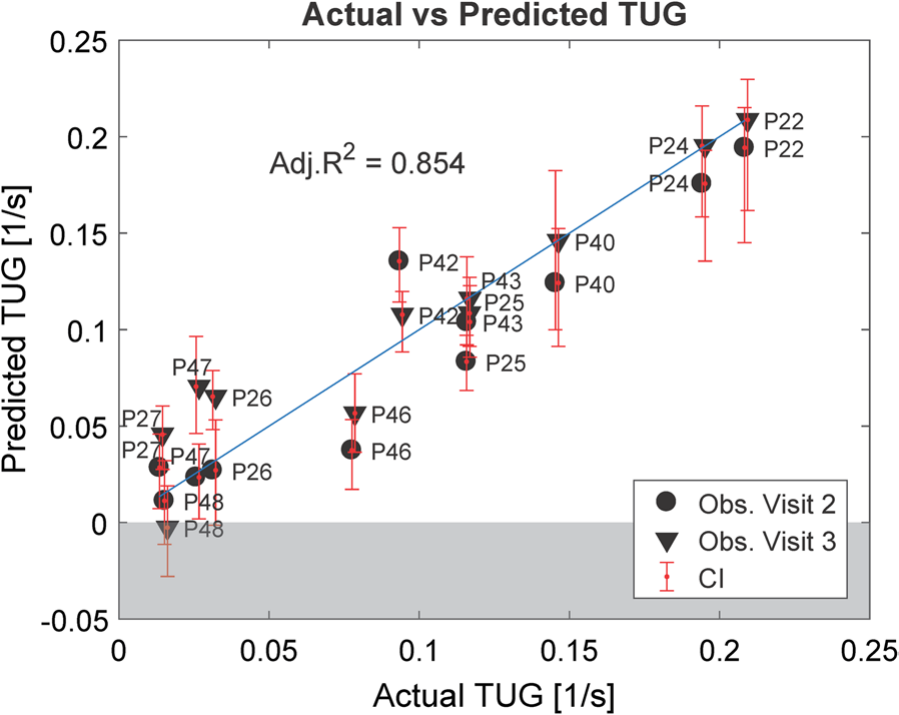
Prediction of TUG based on force and AAN data: unregularized linear model for predicting TUG. Data are taken from subjects that could perform the TUG (n = 11). Predictors: LK_KF and K knee TS. In blue, the identity line is shown

We created, therefore, one model using LF_KF and K knee TS as predictors and one using LF_HF and K knee TS. Compared with the model using only the K knee TS (Fig. 7), both models perform better (LF_KF: average PI = 0.106 s^−1^, Adj. R^2^ = 0.854 (CI = [0.622, 0.976]); LF_HF: average PI = 0.134 s^−1^, Adj. R^2^ = 0.796 (CI = [0.590, 0.954])). The coefficients of LF_KF and LF_HF were positive (LF_KF: β = 0.146, CI = [0.053, 0.203]; LF_HF: β = 0.185, CI = [0.032, 0.288]) and statistically significant (p < 0.001), meaning that higher force leads to better performance in the TUG (since we predicted TUG^−1^). We chose the model with LF_KF due to its smaller PI and higher Adj. R^2^ (Fig. 9).

## Are the Lokomat AAN outcome measures reliable?

We studied the reliability of the AAN outcome measure selected in "Do force measures contribute to the prediction of walking ability?" by comparing the results collected in two sessions executed within 7 days. The sessions were performed by the same examiner (intra-rater reliability). It is essential to determine the consistency of the measures in different sessions to determine whether the measurement error is acceptable for practical applications. We examined both the relative reliability and the absolute reliability of the measure. Relative reliability refers to the degree to which individuals maintain their position in a sample over repeated measurements [13] and it can be measured with the Spearman correlation coefficient [44]. Absolute reliability refers to the degree to which repeated measurements vary for individuals, irrespective of their ranks in a sample [44], and it can be measured with the Bland–Altman plot and the 95% Limits of Agreement (LOAs) [44, 45]. The Bland–Altman plot shows the mean of the two measures plotted against their difference and it can be used to examine the presence of systematic bias and the magnitude of the error compared to the mean value of the measure. The presence of systematic bias is tested with a t-test. The LOAs indicate the range where, for a new individual from the studied population, the difference between any two tests will lie within a 95% probability [44]. If the test is administered to the same individual to detect changes between sessions, these changes are considered significant only if they fall outside the LOAs. Therefore, the LOAs are strictly related to the minimal detectable change (MDC) of a test [13]. We examined the Bland–Altman plot for the predictor selected during the Bolasso procedure (K knee TS) using the free package *BlandAltman* in Matlab [46]. Only ambulatory patients were included in the analysis.

### Results

#### Relative and absolute reliability of K knee TS

**Fig. 10:**
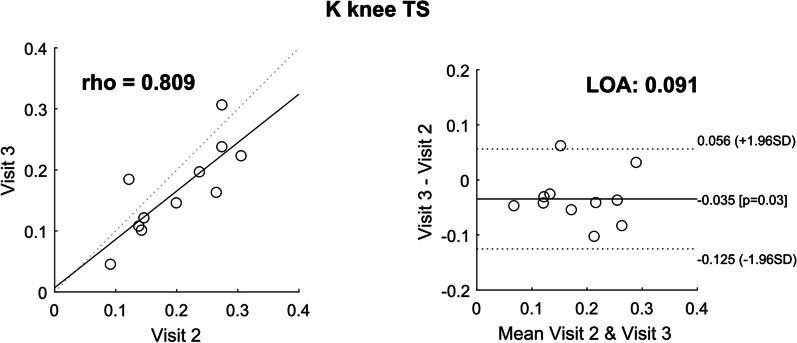
Test–retest reliability: correlation and Bland–Altman plot for K knee TS in ambulatory patients. Rho: Spearman’s coefficient, LOA: Limits of Agreement

The relative reliability of K knee TS in ambulatory patients was good (ρ = 0.809), and the LOAs (absolute reliability) indicate that any change smaller than 0.091 cannot be considered significant (Fig. 10). To give an idea of what this value means in terms of prediction, we calculated the interval created by such LOAs when K knee TS was used in the models to predict the 10MWT and TUG. The LOAs of the K knee TS resulted in an interval of ± 0.722 m/s in the 10MWT and to an interval of ± 0.069 s^−1^ in the TUG^−1^ (corresponding to 14.395 s). Note that this estimation did not consider the uncertainty in the model coefficients. The t-test for systematic bias indicated that the observations of K knee TS from the second measurement (Visit 3) were slightly but significantly lower than the observations from the first measurement (Δ = − 0.035, p = 0.03).

## Can the prediction of walking ability be extrapolated to non-ambulatory and to able-body individuals?

After identifying the Lokomat variables able to provide more information on the walking function of the ambulatory subjects (K knee TS and LF_HF) in "are the Lokomat AAN outcome measures reliable?", we studied if walking speed in the 10MWT could be predicted by the same model also in non-ambulatory subjects and in unimpaired subjects. In the case of the four subjects who could not perform the test, we wanted to check if we could obtain a ‘virtual 10MWT’, which could represent an indication of how close they are to regain some walking function. We applied, therefore, the model generated from ambulatory subjects to predict the 10MWT, using K knee TS and LF_HF as predictors, to all the subjects included in the study (ambulatory and non-ambulatory patients, able-bodied control subjects).

Furthermore, exploratively, we identified among all the AAN outcome measures those which clearly distinguish ambulatory from non-ambulatory patients. We created boxplots for the ambulatory subjects’ data. We then calculated the median of all the outcome measures in the non-ambulatory subjects. We selected the variables in which the non-ambulatory subjects had a median higher than ${q}_{3}+1.5\cdot ({q}_{3}-{q}_{1})$—with q_1_ and q_3_ first and third quartile—of the distribution generated from the ambulatory subjects’ data. Having only 4 non-ambulatory subjects, we limited our analysis to the observation of how their data differ from those of the patients that could walk overground.

We then used the same method described in Table 5 to identify which predictors explain better the 10MWT in able-bodied subjects, including both AAN outcome measures and L-FORCE measures as possible predictors.

### 6.1. Results

#### Can the prediction of walking ability be extrapolated to non-ambulatory and able-bodied individuals?

**Fig. 11:**
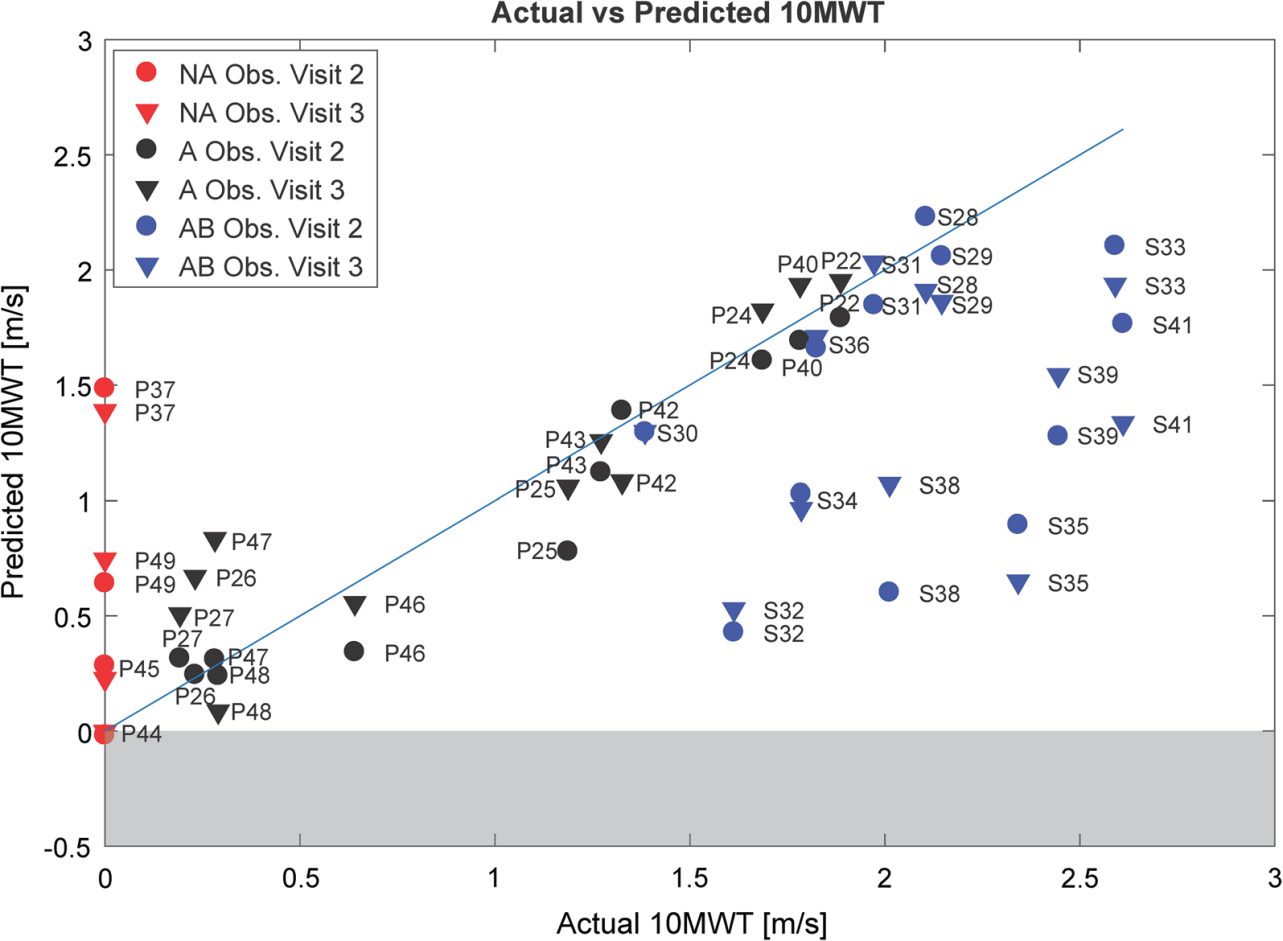
Prediction of "virtual 10MWT" of (i) non-ambulatory subjects (red markers, “NA”), (ii) 10MWT of ambulatory patients (black markers, “A”), and (iii) able-bodied control subjects (blue markers, “AB”). Each subject has one single value of Actual 10MWT (only one test was conducted) corresponding to two values of predicted 10MWT, as they are based on the Lokomat data from the first observation (circle) or the second observation (triangle). The identity line is shown in blue. The model used has 2 predictors (K knee term swing and LF HF) and it was generated from the data of ambulatory patients in "Relative and absolute reliability of K knee TS"

The prediction of 10MWT based on the variables K knee TS and LF_HF for all the subjects participating in the study is shown in Fig. 11 (see Additional file 1: Fig. A.1.6. for the predicted TUG).

In the case of non-ambulatory patients, it seems that the model missed some important information to correctly assign to the non-ambulatory patients a speed close to 0 (or virtually lower than 0), as we had expected: P37, in particular, shows a predicted speed in the range of the less severely impaired patients and of healthy walking. K knee TS and LF_HF were not sufficient to describe the level of function in non-ambulatory patients.

#### Are there other variables that may help to distinguish between ambulatory and non-ambulatory patients?

**Fig. 12:**
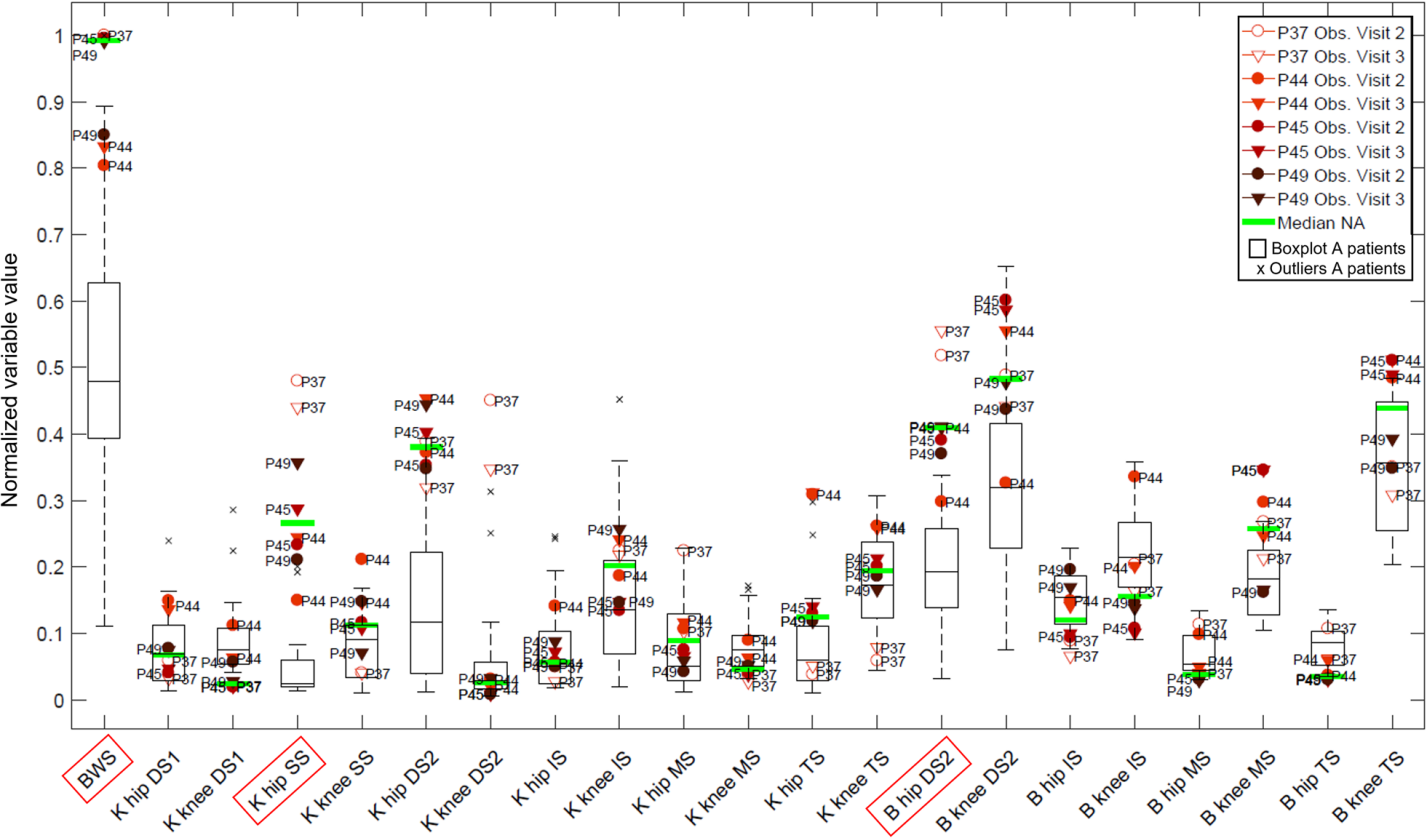
AAN data in all subjects with SCI: boxplots for each variable (BWS: Body Weight Support; K: Stiffness; B: Damping) show the distribution for each variable in ambulatory (A) subjects (boxplots). Observations from the four non-ambulatory (NA) subjects are shown in different shades of red. The highlighted variables are those in which the median of the data of the non-ambulatory patients was higher than the q3 + 3/2 IQR of the data of the ambulatory subjects

We looked, therefore, at the other AAN outcome measures to identify in which ones these patients showed a marked difference from the ambulatory subjects (Fig. 12). Two main phases showed a clear separation between the groups: the single stance phase (BWS, K hip SS) and the second double support phase (B hip DS2).

The non-ambulatory patients needed a BWS higher than 64% of their body weight (BWS was normalized by the maximum achievable BWS, equal to 80% of the body weight). For the other variables mentioned above, the non-ambulatory patients showed a residual support in a higher range of values compared to the other patients. However, given the very few observations collected, it is not possible to show in this study if these variables would be able to correctly assess the level of function within the non-ambulatory subjects.

#### Can the model predict walking speed in able-bodied subjects?

**Fig. 13:**
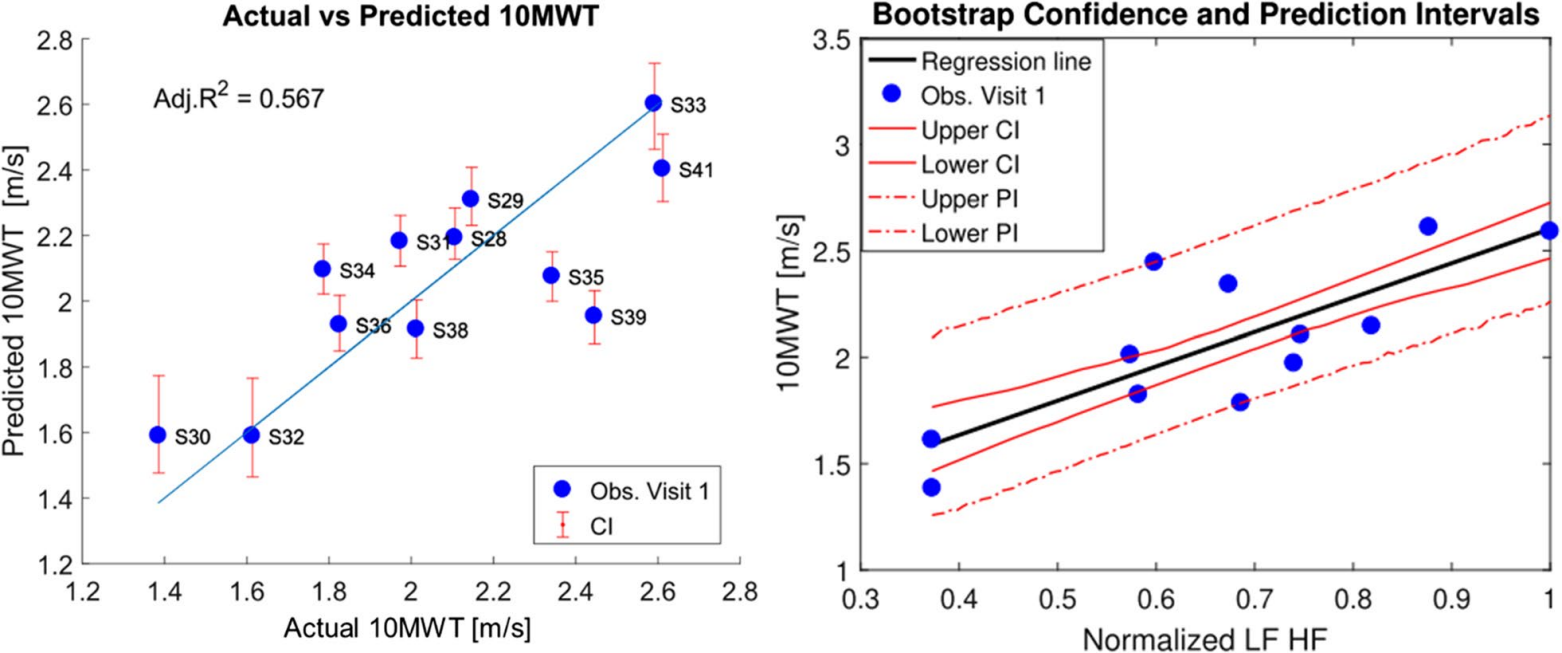
Prediction of 10MWT in able-bodied subjects: prediction of 10MWT in AB subjects using LF_HF as only predictor

The models created from patients’ data with one predictor (K knee TS) and two predictors (K knee TS and LF_HF) performed very poorly in predicting the 10MWT in able-bodied subjects (Fig. 11). Consequently, to explore which variable can predict gait speed in unimpaired subjects, the same procedure (Table 5) was applied to these data. L-FORCE at Hip Flexion was chosen 63.5% of the times (see Additional file 1: Fig. A.1.5.) and it was found to be a significant predictor of walking speed in able-bodied subjects (β = 1.612 (CI = [1.169, 1.888]), p = 0.002, Adj. R^2^ = 0.567 (CI = [0.225, 0.826])). The model average PI is 0.834 m/s. Bolasso selected as first most frequent predictors the four L-FORCE measures (Fig. 13).

## Discussion

*The AAN controller in a robotic gait trainer is feasible for individuals with SCI*

We investigated the use of a robotic trainer (Lokomat) as a tool to assess ‘walking ability’ in individuals with Spinal Cord Injury (SCI). We showed the relationship between data measured with a robotic assist-as-needed (AAN) control and clinical scores related to walking ability: 10-Meter Walking Test (10MWT) and Timed Up and Go (TUG). We also examined how measures of isometric joint torques relate to the walking assessments. The AAN-based protocol tested in this study could be performed by ambulatory and non-ambulatory individuals with complete and incomplete spinal cord injury (SCI) during training. The isometric joint torques could be measured with the same device. The task, as shown by the NASA-TLX results ("what are the most representative robotic variables that explain walking ability?"), did not frustrate nor required excessive effort from the subjects, who showed a rather high perceived performance and low mental and physical demand. Therefore, we can conclude that the approach is feasible to be implemented in clinical practice.

One of the concerns that many clinicians have about robotic gait trainers is that the environment where the people train is too artificial and different from overground walking, and the support provided by the device may mask what the patient can actively do; consequently, the assessments performed in a robotic gait trainer lack face validity according to their view. We showed, instead, that it is possible to measure in a robotic gait trainer functions that are related to overground performance in walking tests such as the 10MWT and TUG.

*The support required at terminal swing (K knee TS) is the most representative robotic variable that explains walking ability*

In "Do force measures contribute to the prediction of walking ability?", we identified an informative predictor of overground walking speed in ambulatory patients, when training in the Lokomat: the support (stiffness of the impedance controller) required from the robotic exoskeleton at the knee during terminal swing (K knee TS). This predictor was consistently selected during the bootstrap procedure and, alone, it could be used to generate a model able to explain 74% of the variance in the 10MWT data and 61% in the TUG data.

The relevance of terminal swing was already suggested from the technical validation experiment that we performed in a previous study [21]. There, we simulated different levels of weakness on a robotic test bench, and we studied how the AAN controller would react to them. We observed that the stiffness increased proportionally to the level of simulated weakness, but only in two phases of the gait cycle: on the transition between stance and swing and at terminal swing. With human subjects, while walking in the Lokomat, only the support required at terminal swing seems to be related to the level of impairment of the patient. In gait literature, the importance of terminal swing is highlighted by the fact that a smooth transition from swing to stance and an adequate step length are considered two requirements of functional gait [27, 47, 48]. In EMG studies, it was shown that a burst of muscle activity at the quadriceps is required to complete knee extension before heel strike [26, 27]. The AAN algorithm implemented a deadband around the reference trajectory to allow for some deviations (Additional file 3: Fig. A.3.1); the patient had, therefore, the possibility and the necessity to control foot placement and step length at terminal swing. The increased support required in this phase was likely needed to extend adequately the knee before foot contact. It is possible, however, that other predictors would be chosen if a different patient population was tested (e.g. more severely impaired patients).*Isometric force measures contribute to the prediction of walking ability.*

The assessment of isometric joint torque ("are the Lokomat AAN outcome measures reliable?") added an important predictor to the model and improved the accuracy of prediction of speed of both tests (ΔR^2^ 10MWT = 0.119, ΔR^2^ TUG = 0.248), especially in the TUG, where either the maximum isometric knee flexion torque or the maximum isometric hip flexion torque seemed to be even more important than the predictor K knee TS. The TUG includes standing up from a chair, turning and sitting down. Despite being highly correlated with the 10MWT [29], the TUG requires other factors to be completed successfully, such as balance and force [49, 50]. It is reasonable, therefore, that information related to muscle force highly improved the prediction of the TUG. In literature, lower limb muscle strength was associated with longer sit-to-walk duration in the TUG in elderly subjects (knee extensors [50]) and in stroke patients (affected ankle plantar flexors [51]), however in those studies the hip and knee flexors were not tested.

The ability to walk is a composite, multifactorial construct consisting of factors such as motor control and coordination, muscle strength, balance and posture, range of motion, proprioception and muscle tone [41]. In the 10MWT prediction, the first variable of muscle force chosen by the regression procedure was the maximum hip flexion isometric torque. This is in line with literature, where the strength of the hip flexors at the less affected side has been found to correlate well with gait speed as measured by the 10MWT [42]. A study using neuromusculoskeletal model of gait also found that gait performance is most affected by weakness at the hip flexors (together with ankle plantar flexors and hip abductors) [52].

It can be hypothesized that the performance measured in the AAN-based assessment is mainly due to the ability of modulating motion and force, rather than to the ability of applying high forces. The task of following a reference trajectory with the ankle requires a timely and accurate coordination of the hip and knee joints and the ability to process the visual information displayed on the screen while providing the correct motor commands. In order to walk, we need, however, more than being able to control motion—force modulation is undoubtedly one of the other main components of walking function [41]. The isometric torque assessment seemed, thus, a good complementary assessment to the AAN-based assessment.

*The support required at terminal swing (K knee TS) has good relative reliability but poor absolute reliability*

The Limits of Agreement ("Can the prediction of walking ability be extrapolated to non-ambulatory and able-bodied individuals?", Fig. 10) indicate that a change in the measurement can be considered true only if it falls outside these limits [44]. Therefore, a difference of 0.091 in normalized K knee TS will be necessary to show a significant improvement in the test. Considering that the maximum normalized stiffness is 1, this difference corresponds to 9.1% change in stiffness. From literature, we know that the Minimal Detectable Change in SCI in the 10MWT is 0.13 m/s and in the TUG is 10.8 s [2]. Our intervals determined from the reliability of the predictor K knee TS are higher (10MWT: 0.72 m/s, TUG: 14.4 s). However, the aim of our study is not to replace the standard timed tests, but rather to use the timed tests as a reference to identify the variables measured in the Lokomat that can provide more information on the patient’s walking ability. The significant difference between the two visits may indicate the presence of a learning effect from the first to the second measurement. Interestingly, the Spearman correlation between the AAN outcome measured in Visit 2 and 3 was rather high (ρ = 0.809), confirming how the high inter-subject variability masked the intra-subject error [53] and showing once more how important it is to consider both the relative and absolute reliability when validating a new measurement tool.

With regards to the reliability of L-FORCE, we refer to the work of Bolliger et al. [31], where the L-FORCE showed a fair to good reliability (intra-rater reliability for LF_HF and LF_KF ranged from 0.50 to 0.91; the SEM ranged between 6.5 Nm and 11.6 Nm for single measures).

*The prediction of walking speed in the 10MWT based on K knee TS cannot be extrapolated to non-ambulatory nor to able-bodied individuals*

In Sect. "discussion", we applied the models generated in Sect. "Are the Lokomat AAN outcome measures reliable?" to predict a ‘virtual 10MWT’ in non-ambulatory patients. This value could represent an indication of how close they are to regain some walking function. However, this model was not able to correctly predict function in people that cannot walk overground. Since the model does not consider variables related to the stance phase, it overestimates the ‘virtual walking ability’ of the subjects given their residual function measured during swing phase in the Lokomat. The variable K knee TS reflects the guidance at terminal swing required in the Lokomat to extend the knee right before foot placement. While examining case by case the four non-ambulatory patients, we can see that P37 had a high knee extension isometric torque (LF_KE) and MMT = 5 for both knee extensors: he was therefore able to extend the knee actively at the end of swing phase and required low robotic support. For this reason, this patient scored high on the ‘virtual 10MWT’. The prediction of 10MWT for the other subjects was in the range of the wheelchair-dependent walkers (P44), supervised/indoor walkers (P45) and walkers with aids (P49) [54]. P44 and P45 would be classified as unable to walk independently in the community (according to the cutoff speed of 0.59 m/s determined in [55]). Indeed, P45 had a complete lesion at T10 and no motor function below the lesion level. P44, despite being classified as ASIA D, was at an advanced stage of Multiple Sclerosis and he/she was not able to stand or walk. P49, despite not being able to exert force at the knee joint, had some residual motor function at the hip flexors level (MMT hip flexors = 3), therefore scoring relatively high on the ‘virtual 10MWT’, which was predicted based also on the maximum hip flexor torque.

### Variables related to stance phase and to push-off phase may help distinguish ambulatory from non-ambulatory subjects

There are likely other important predictors for gait-related functions in the non-ambulatory population and some of them could be measured in a robotic gait trainer, as suggested in Fig. 12. Measures relative to the stance phase (and therefore to the ability of support the body weight) and push-off phase could be good candidates to investigate, as they may also help differentiating between non-ambulatory patients with different level of function. The support of the body is one of the main determinants of gait [26, 27]. If this condition is not met, the importance of other functions, such as the ability to place the foot correctly at the end of swing, is negligible. At push-off most of the power during gait cycle is generated [26, 27]. The conditions at push-off, especially the rate of knee flexion, determine the knee flexion peak during swing phase and the consequent ability to clear the ground during swing [56]. The main muscles that influence knee flexion velocity during the double support phase are the gastrocnemius (ankle plantarflexor) and iliopsoas (hip flexor) [57]. However, due to the use of foot-lifters in the Lokomat, the activation of the gastrocnemius is partly reduced [58–60]. This requires, as compensation, to produce an increased hip flexion rate and quadriceps EMG activity at the end of stance phase to lift the foot above the treadmill [59, 61]. While the less impaired patients were able to cope with this demand, the more severely affected patients could not reach the knee flexion velocity required by the Lokomat during the double support phase, as suggested by the high residual damping required at DS2. This phase was already highlighted in our previous study performed with a robotic test bench: the higher the simulated impairment, the higher the support required during the preparation of swing phase [21].

We believe that, if more data from non-ambulatory subjects with different level of function were collected, it would be possible to generate a model (possibly adding one or two other predictors) that explains continuously the ‘walking ability’. In non-ambulatory patients, this assessment would show how close they are to recover walking. However, it would be challenging to validate this assessment, since we would need a way to measure how close non-ambulatory subjects are to regain some walking function. Possibly, a study in acute and sub-acute patients, rather than chronic, will help address this question. One could follow the same patients longitudinally and see if improvements in functional and impairment scales are paralleled by improvements in the AAN outcome measures.

### Among the Lokomat data, isometric force is the best predictor of walking speed in able-bodied subjects

It is also not possible to explain the 10MWT of able-bodied subjects with the AAN outcome measures proposed here. The fact that unimpaired subjects did not conform to the model suited for the ambulatory patients confirms that the AAN-based assessment is affected by the underlying impairment of the subjects, and it is not capturing individual speed variations in unimpaired individuals. Moreover, able-bodied subjects often try to impose their own foot trajectory while walking in the Lokomat, thereby deviating from the reference trajectory programmed within the device. While the deadband implemented in this adaptive algorithm tried to address this problem, it may be insufficient to accommodate completely the physiological variability in gait pattern expressed by able-bodied subjects. The same is true for patients with a mild impairment in walking ability, whose walking speed is close or equal to normal; the AAN-based assessment is likely not suitable for them.

The main predictor selected in the SCI population (K knee TS) was specific for the impairment, while the isometric torque at hip flexion seemed to be an important predictor of speed both in the ambulatory SCI and able-bodied populations. There is evidence in literature that maximum isometric hip joint torque is a significant predictor of gait speed in people after stroke [62] and SCI [42]. In able-bodied subjects, there is some evidence that lower limb muscle strength correlates with gait speed [63–65].

### Limitations and challenges

Despite being able to identify two good predictors of walking speed (robotic stiffness required at terminal swing and isometric hip flexor force), we cannot state, with the available data, that the robotic assist-as-needed control as we implemented it in the Lokomat is valid and reliable enough to be used as assessment of ‘walking ability’ in the clinic. Further work is needed to translate these techniques into clinical practice.

The intra-rater reliability between two sessions needs to be improved before the test can be used in therapy. Several factors may have affected the reliability of the measures. First, a learning effect between sessions was still present despite having included in the protocol a first session of familiarization. Second, even if we tried to install the subject in the Lokomat always in the same way and to use always the same hardware and software settings across sessions, it is challenging to have perfectly reproducible conditions. Third, the AAN-based assessment relied on subjective attention and concentration during the task, resulting in a perceived higher mental rather than physical demand in the NASA-TLX. It is likely that cognitive and visual aspects had an influence on the outcomes, as already noticed in literature [66]. Lastly, the reference gait trajectory used in the study was subjectively adapted to the subject’s individual gait pattern in the first visit, according to the experience of the examiner who performed the test. It may be that the additional challenge of following a gait pattern different from one’s own resulted in a more variable performance that negatively impacted the validity and the reliability of the assessment. Based on these considerations, more practice should be allowed before performing the assessment and a personalized reference gait trajectory should be determined with objective methods. The trajectory could be defined based on anthropometric measures [67] or on database-driven methods, taking a pool of physiological trajectories as templates [68]. Alternatively, the error metric for the adaptation of the impedance, which is now based on the kinematic deviation between reference and actual trajectory, could be based, for example, on the success in different sub-tasks of walking (e.g. stability in stance, foot placement) [69]. Clinicians should also be aware that adapting the reference trajectory from session to session is also likely to negatively affect the reliability of the assessment.

The choice of unequal thresholds’ width may have impacted differently the performance of the participants in the different gait phases: as shown in the Additional file 3, thresholds width varied throughout the gait cycle as it was narrower in correspondence of critical gait tasks (e.g. terminal swing for foot placement). Narrow thresholds require a more precise tracking of the reference trajectory and may have increased the challenge of the task in those gait phases, potentially leading to a higher likelihood of the corresponding AAN parameters to be chosen as predictors of walking ability, as it may have happened for the knee stiffness at terminal swing.

The adaptation algorithm theoretically adapted the GF in a range between 0 and 100%. However, we saw that, depending on the weight of the patient’s limbs, on the severity of impairment and on the BWS value, a value of GF much lower than the maximum is sufficient to walk in the Lokomat. The highest hip and knee GF required by a subject in our study, average during the whole gait cycle, was 20% (with a BWS equal to 80% of the body weight). This means that the actual range where the subjects can show the impairment is limited and meaningful information can only be seen after the GF has reached this low value (after 15 steps).

We could not develop a model to predict the walking-related functions of the patients continuously from the non-ambulatory to the ambulatory phase. More data from non-ambulatory subjects with different level of severity needs to be collected to see if the variables identified in Fig. 12 are meaningful predictors of function in severely and moderately affected patients. A longitudinal study in patients with acute or sub-acute lesions should be carried out to understand how the AAN outcome measures change over time in the same patients. Studying the recovery within the same patients will show if individual changes can be measured by the AAN-based assessment. Other populations with neurological impairments need to be tested to check if the predictors of walking speed identified for SCI are applicable also to other pathologies.

One important question that this study raises is how to validate novel objective assessments. We faced the conundrum of trying to validate an assessment that claims it can be more objective than existing assessments, by comparing it against the same standard assessments that we were trying to improve. The 10MWT and TUG had a clear floor effect that prevented to assess people that could not walk. The other scores (WISCI II, BBS, FAC, MMT, AIS) were ordinal-based and too coarse-grained to be directly compared with the Lokomat measures. We followed standard practice for clinical validation studies, but we believe future studies should incorporate complementary validation techniques. For example, one should either include in the validation study more sophisticated assessments of gait (e.g. motion capture gait analysis) or, as we demonstrate in [21], develop a controlled environment to systematically simulate known neuromotor impairments and study if the novel assessment can correctly measure them.

Lastly, the model used to predict the walking tests is a simple linear regression: it may be that a generalized linear model with another link function would lead to better predictions. The simulation we performed in [21] showed that there are factors (such as increased joint stiffness) that cause a non-linear relationship between impairment and AAN-determined level of support. Also, the interaction between different variables, especially between the BWS and the other AAN outcome measures needs to be better explored.

### Implications for walking training paradigms

The identification of certain predictors for speed of walking overground leads to the question: can individuals improve their walking speed by training the functions underlined by the predictor variables (i.e. foot placement at terminal swing and hip force)? This question deserves further investigation, but it is beyond the scope of this study. Functions such as foot placement at terminal swing or support during stance phase could be trained with the help of robots that allow setting different levels of support throughout the gait cycle and visualizing on the screen the performance for proper feedback to the patient and therapist. At the same time, these functions can be assessed with such robotic controllers and their relationship with walking ability overground explored. We can also hypothesize that, during walking rehabilitation, a certain sequence in the required improvement of walking-related functions exists: severe patients might need to focus first on support during stance, while the more they progress, tasks requiring motor control and precise placement of the foot might become more important.

Robotic gait trainers allow the therapists to adapt the support parameters (e.g. limb guidance and body weight support) manually to challenge the patient in an optimal way. However, these functions are not often used in clinical practice because clear guidelines on the progression of these parameters are lacking and the consequences of the changes on the therapeutic outcomes are not clear. Adaptive algorithms, such as the one developed for this study, can be used safely for determining the optimal level of assistance for every patient. Positive experiences with adaptive controllers for walking training of neurological patients have been shown also in other groups [70, 71]. Fricke et al. [70], in particular, compared manual tuning of robotic assistance with automatic tuning and found out that automatic tuning is faster in reaching stable levels of robotic assistance and provides lower level of assistance, which in turn could lead to increase patient challenge and to better training outcomes [72]. Although there are many challenges in translating these techniques into commercial devices [73], we have demonstrated that our approach, as implemented in this study, was safe at all times, and all the individuals with SCI could use it and reported a high perceived performance (Sect. "What are the most representative robotic variables that explain walking ability?") and no frustration. Therefore, this approach has been integrated as assist-as-needed *training* modality, rather than as assessment, in the Lokomat^®^Pro as of 2020 [74]. Adequate challenge of the patient and improved training outcomes would need to be investigated in future studies.

## Conclusion

We showed in this study that a single variable resulting from the proposed adaptive controller, that was measured during training in the Lokomat (the support required by the knee at terminal swing—K knee TS) can explain most of the variance of the timed walking tests. The additional consideration of an isometric force measure collected in a specific test available in the Lokomat (L-FORCE) makes the explained variance of the models increase above 85%. While our study concludes that the current implementation is not ready for assessment in clinical practice, these results are very promising because they show that walking ability can be measured in a robotic gait trainer in a safe and efficient way, and individuals with Spinal Cord Injury have a good acceptance of the approach, which demonstrated its feasibility also as a training modality. Further efforts should improve the model to predict the clinical scores from the AAN outcome measures, extending this also to patients that cannot walk yet, and increase the reliability of the measures. We hope that our results and recommendations will help reaching the long-term goal of developing a valid and reliable assessment that, with a standardized protocol and few measures, can assess walking ability in patients will all levels of severity and be quickly and easily administered during training. Accessible assessments mean personalized therapy, possibility of demonstrating improvements to insurances and increased patient’s motivation with positive effects on his/her recovery.

### Supplementary Information

**Additional file 1.** Selection of predictors in Bolasso.

**Additional file 2.** Corralations between NASA Task Load Index (TLX) questionnaire responses and clinical scores (10MWT, TUG, WISCI).

**Additional file 3.** Implementation and parameters used in the Assist-as-Needed controller.

## Data Availability

The datasets used and/or analyzed during the current study are available from the corresponding author upon reasonable request.

## Footnotes

1 https://knowledge.hocoma.com/posttraining/safe-walk/?product_name=lokomat.
