# Supplementary material for: Assessing walking ability using a robotic gait trainer: opportunities and limitations of assist-as-needed control in spinal cord injury
Source: J Neuroeng Rehabil. 2023 Sep 21;20:121. doi: 10.1186/s12984-023-01226-4 (PMC10515081; doi:10.1186/s12984-023-01226-4)
Supplement: Supplementary file 1 — Additional file 1. Selection of predictors in Bolasso. [file 12984_2023_1226_MOESM1_ESM.docx]

Additional File 1

- 1. Selection of Predictors in Bolasso

Bolasso was used as feature selection method to identify which AAN-outcome measure(s) could better predict the timed walking tests. Here the variables selected in 1000 bootstrap runs are presented.

- - 1. Prediction of 10MWT using only AAN outcome measures


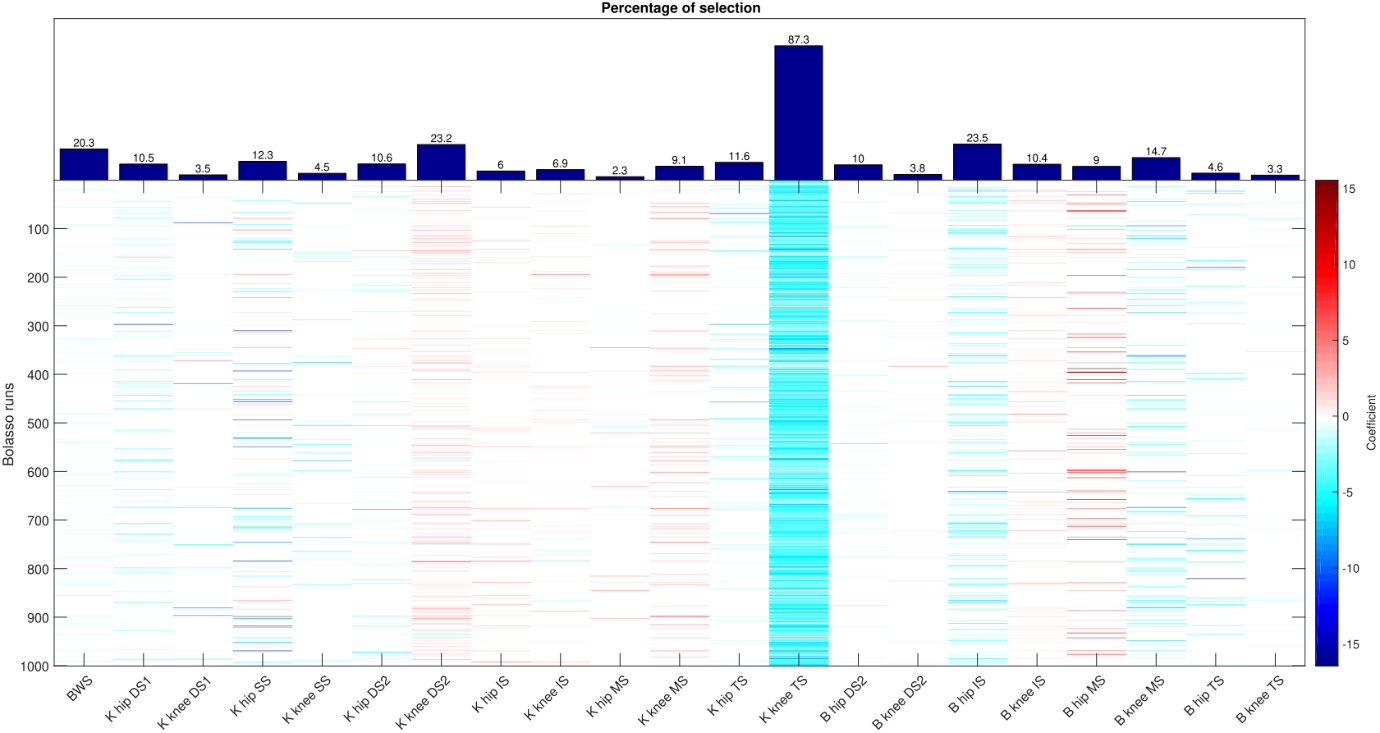


Figure A.1.1.: Output of the Bolasso algorithm for the prediction of 10MWT in ambulatory patients. The colored bars indicate if the coefficient of the predictor in a certain Bolasso run was different from 0, positive (red) or negative (blue). The percentage of selection is shown in the bar plot above.

- - 1. Prediction of TUG using only AAN outcome measures


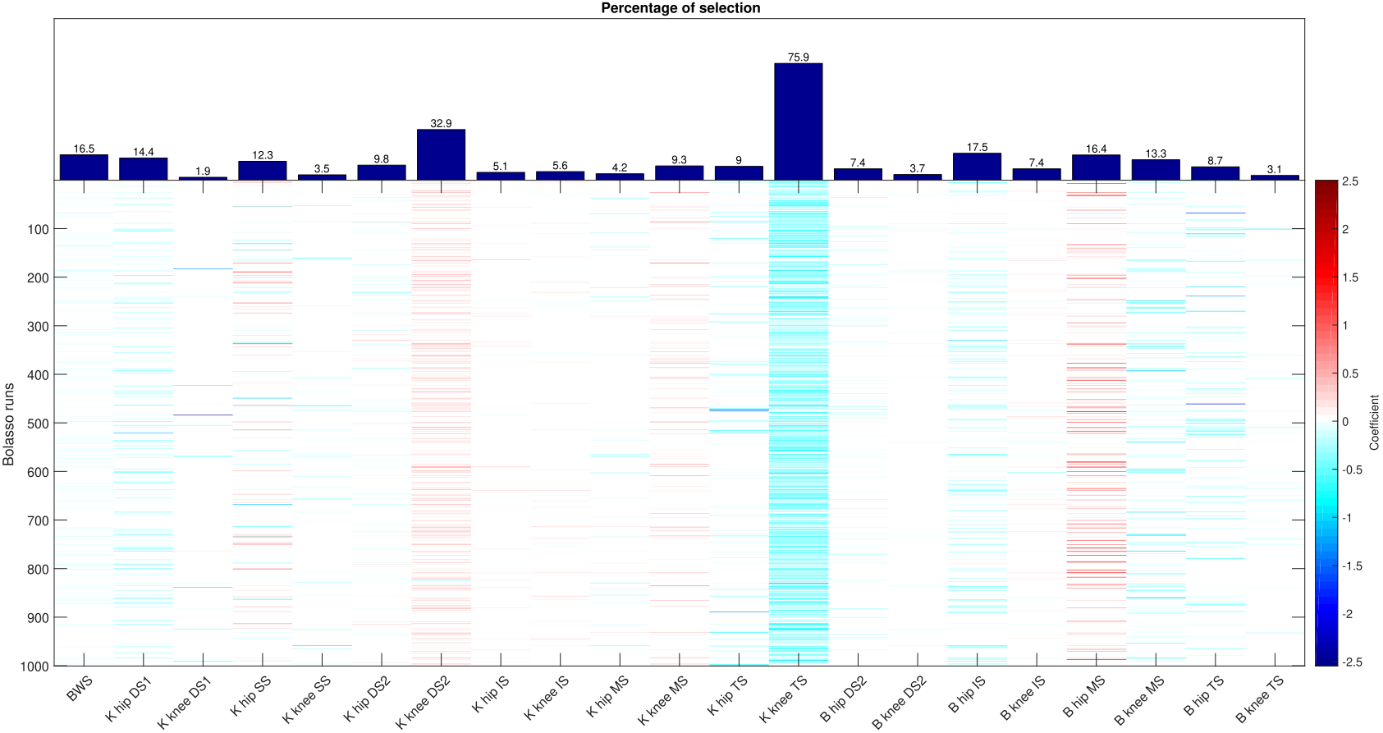


Figure A.1.2.: Output of the Bolasso algorithm for the prediction of TUG in ambulatory patients. The colored bars indicate if the coefficient of the predictor in a certain Bolasso run was different from 0, positive (red) or negative (blue). The percentage of selection is shown in the bar plot above.

- - 1. Prediction of 10MWT using AAN outcome measures and L-FORCE measures


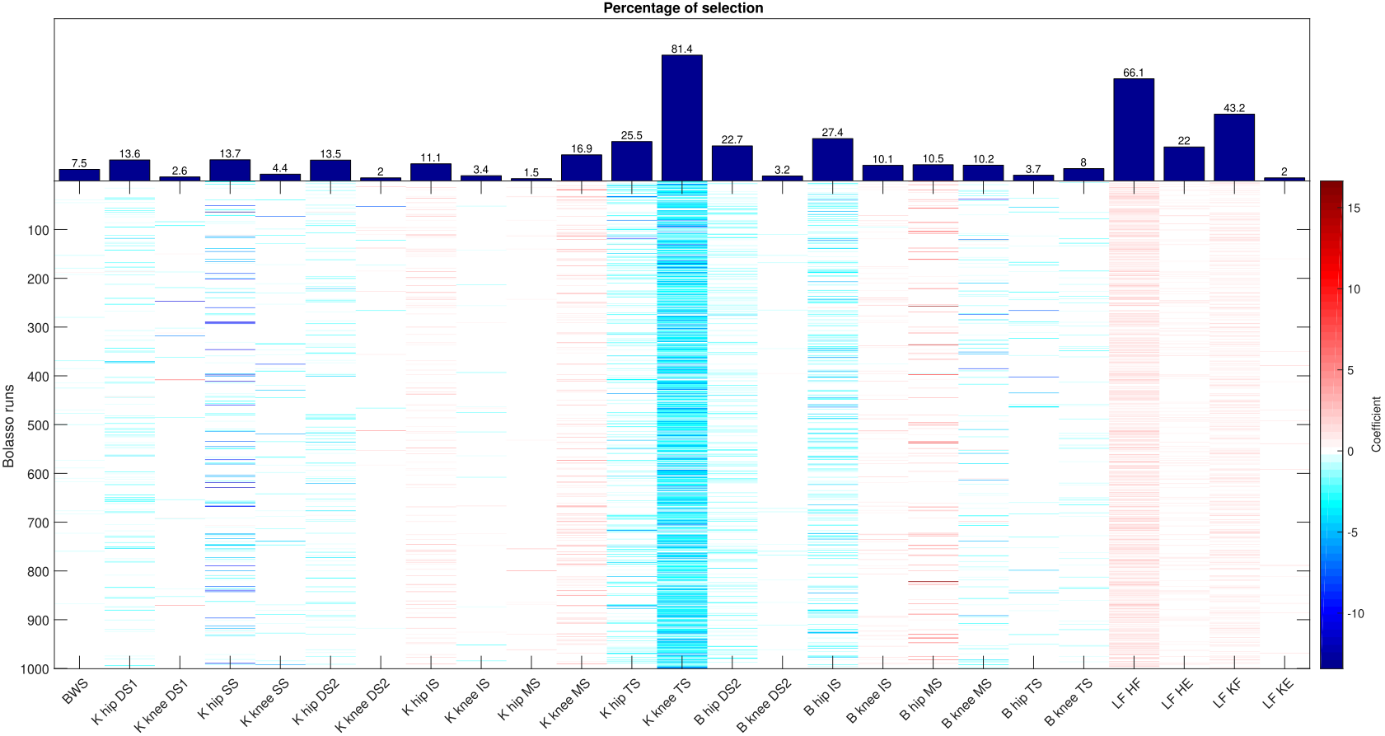


Figure A.1.3.: Output of the Bolasso algorithm for the prediction of 10MWT in ambulatory patients when also the L-FORCE measures are added to the pool of possible predictors. The colored bars indicate if the coefficient of the predictor in a certain Bolasso run was different from 0, positive (red) or negative (blue). The percentage of selection is shown in the bar plot above.

- - 1. Prediction of TUG using AAN outcome measures and L-FORCE measures


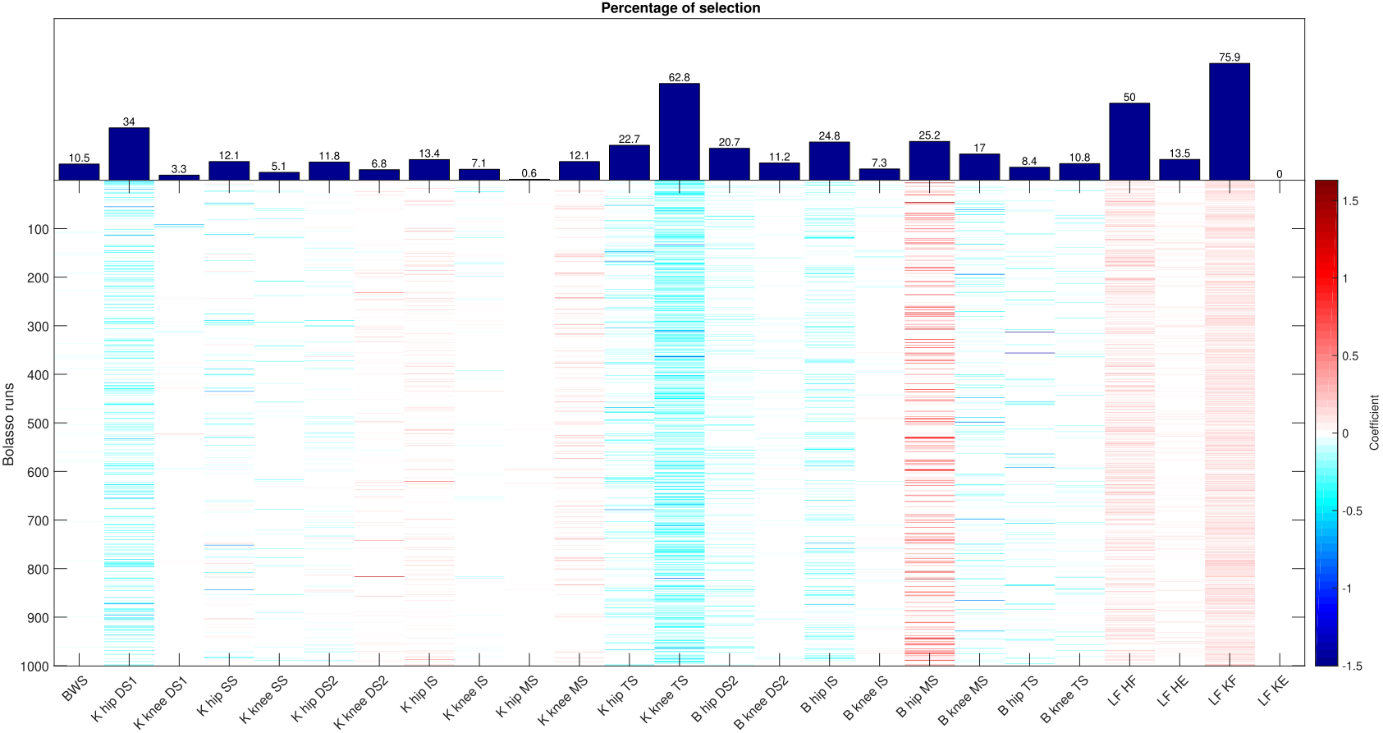


Figure A.1.4.: Output of the Bolasso algorithm for the prediction of TUG in ambulatory patients when also the L-FORCE measures are added to the pool of possible predictors. The colored bars indicate if the coefficient of the predictor in a certain Bolasso run was different from 0, positive (red) or negative (blue). The percentage of selection is shown in the bar plot above.

- - 1. Prediction of 10MWT in able-bodied subjects using AAN outcome measures and L-FORCE measures


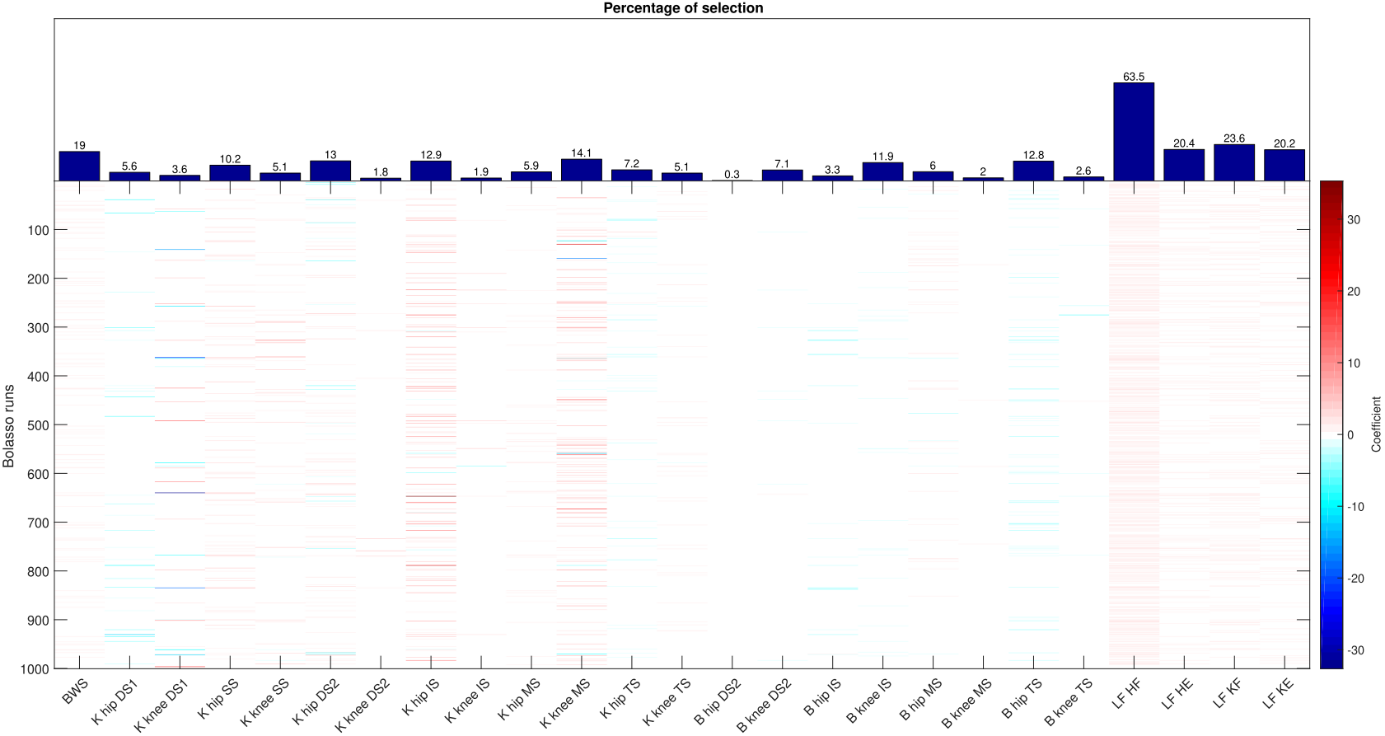


Figure A.1.5.: Output of the Bolasso algorithm for the prediction of 10MWT in able-bodied subjects when also the L-FORCE measures are added to the pool of possible predictors. The colored bars indicate if the coefficient of the predictor in a certain Bolasso run was different from 0, positive (red) or negative (blue). The percentage of selection is shown in the bar plot above.

- 1. Prediction of TUG in Non-ambulatory Patients and in Able-bodied Subjects


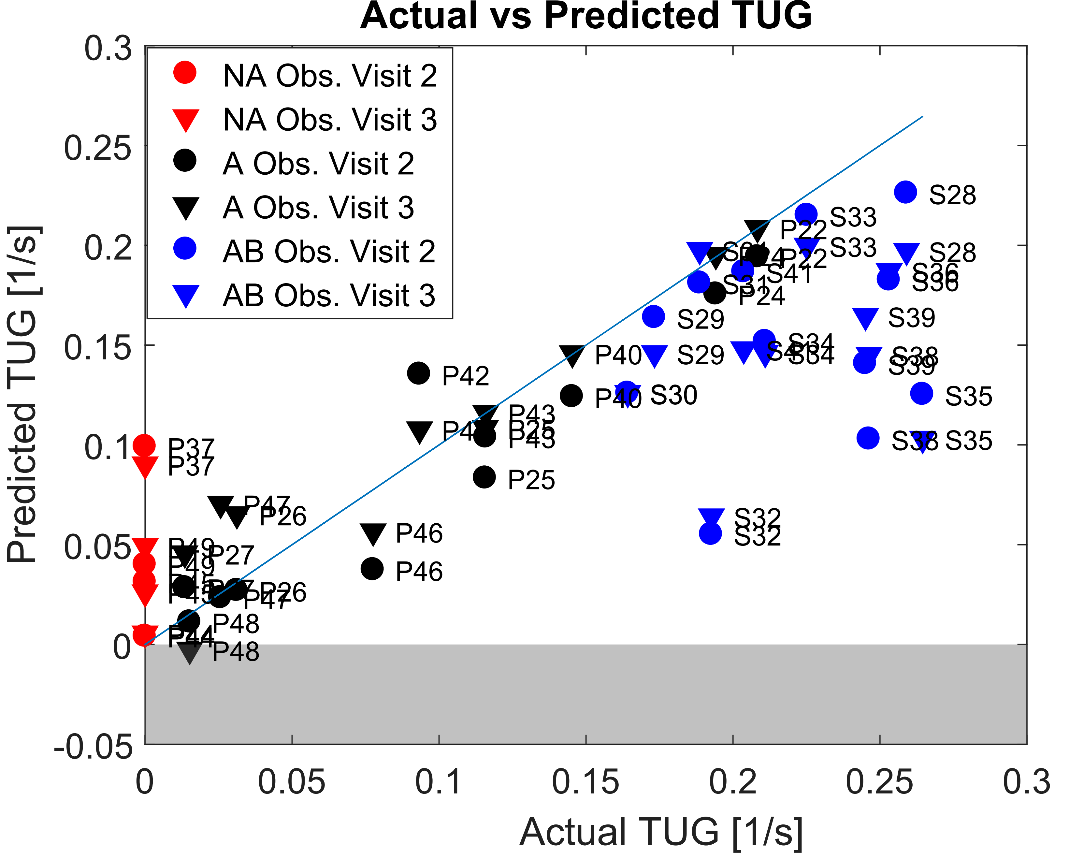


Figure A.1.6.: Prediction of virtual TUG using model with 2 predictors (K knee TS and LF KF). In blue, the identity line is shown
