# Supplementary material for: Assessing walking ability using a robotic gait trainer: opportunities and limitations of assist-as-needed control in spinal cord injury
Source: J Neuroeng Rehabil. 2023 Sep 21;20:121. doi: 10.1186/s12984-023-01226-4 (PMC10515081; doi:10.1186/s12984-023-01226-4)

Additional File 2

At the end of Visit 2 and 3, the participants were asked to fill the NASA Task Load Index (TLX) questionnaire (Table A.2.1).

The correlation between the participants’ answers (only ambulatory patients were considered) and the clinical scores (10MWT, TUG, WISCI) is presented in Figure A.2.1.

| **NASA Task Load Index** | |
| --- | --- |
| Mental Demand | How mentally demanding was the task? |
| 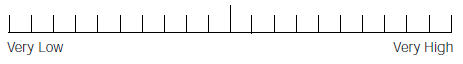 | |
| Physical Demand | How physically demanding was the task? |
| 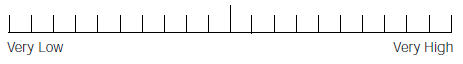 | |
| Temporal Demand | How hurried or rushed was the pace of the task? |
| 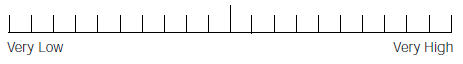 | |
| Performance | How successful were you in accomplishing what you were asked to do? |
| 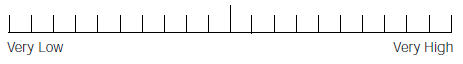 | |
| Effort | How hard did you have to work to accomplish your level of performance? |
| 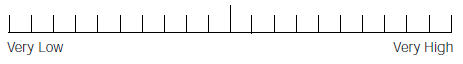 | |
| Frustration | How insecure, discouraged, irritated, stressed, and annoyed were you? |
| 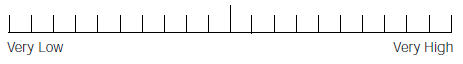 | |

Table A.2.1: NASA TLX questionnaire used in the study. Participants were asked to fill in this questionnaire after performing the AAN-based assessment on both visits.

| Figure A.2.1: Correlation plot between clinical scores (10MWT [m/s], TUG [1/s], WISCI II) and TLX questionnaire collected after the second AAN-based assessment visit (Visit 3). The values indicated the Spearman correlation coefficient. The significant correlations coefficients (p<0.05) are indicated in red. Only ambulatory subjects are included in the analysis. |
| --- |


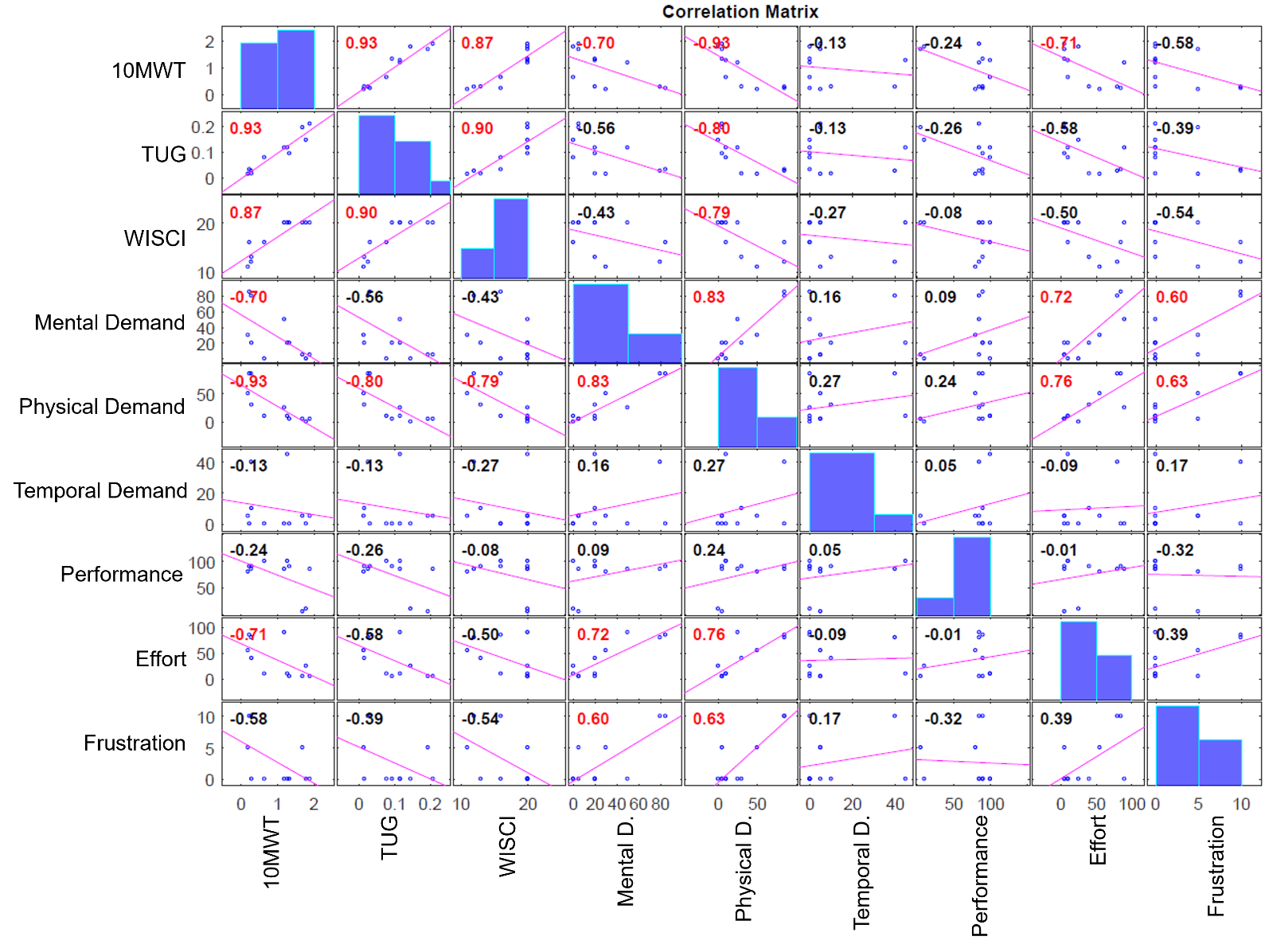

Supplement: Supplementary file 2 — Additional file 2. Corralations between NASA Task Load Index (TLX) questionnaire responses and clinical scores (10MWT, TUG, WISCI). [file 12984_2023_1226_MOESM2_ESM.docx]
