# Supplementary material for: Assessing walking ability using a robotic gait trainer: opportunities and limitations of assist-as-needed control in spinal cord injury
Source: J Neuroeng Rehabil. 2023 Sep 21;20:121. doi: 10.1186/s12984-023-01226-4 (PMC10515081; doi:10.1186/s12984-023-01226-4)
Supplement: Supplementary file 3 — Additional file 3. Implementation and parameters used in the Assist-as-Needed controller. [file 12984_2023_1226_MOESM3_ESM.docx]

Additional File 3

# Assist-as-Needed Controller

We provide here more details on the implementation and the parameters used in the Assist-as-Needed controller.

Equations (1) to (9) include several parameters that were fixed in our experiment and are reported in Table A.3.1.

Parameters of the AAN Controller

| **Parameter** | **Value** | **Unit** |
| --- | --- | --- |
| *K_Hip,1_* | 1200 | Nm/rad |
| *K_Knee,1_* | 900 | Nm/rad |
| *B_Hip,1_* | 55 | Nms/rad |
| *B_Knee,1_* | 36 | Nms/rad |
| *BWS_1_* | 0.7∙*BW* | kg |
| $\gamma_{1}$ | 0.9 | - |
| $\gamma_{2}$ | 0.9 | - |
| $\gamma_{3}$ | 0.95 | - |
| $g_{1}$ | 0.1 | Nm/rad |
| $g_{2}$ | 0.1 | Nms/rad |
| $g_{3}$ | 20 | m^-1^ |
| *A* | 0.6 | - |
| *p_1_* | 0.005 | rad^4^ / N^4^m^4^ |
| *p_2_* | 0.0005 | - |

Table A.3.1: Parameters used in the adaptive controller equation. K_Hip,1,_ K_Knee,1,_ B_Hip,1,_ B_Knee,1,_ BWS_1_: initial values of impedance and BWS; BW: Body Weight; $\gamma_{1,}\gamma_{2,}\gamma_{3}$: forgetting factors. $g_{1,}g_{2,}g_{3}$: error gains. A: steepness of the tanh function. p_1,_ p_2:_ parameters for the BWS error threshold calculation.

Thresholds of maximum allowed deviations (lower $th_{lo}$ and higher $th_{hi}$) are determined around the reference angular trajectory$\boldsymbol{q}_{ref}\left( i \right).$ We defined deadbands ad-hoc to ensure safety in critical phases of the gait cycle (e.g. terminal swing for correct foot placement) and at the same time allow physiological variability in the phases where larger kinematic variations would not result in safety concerns. Thresholds’ width varies within the gait cycle (Figure A.3.1), but the same set of thresholds applies to all the participants to the study.

| A  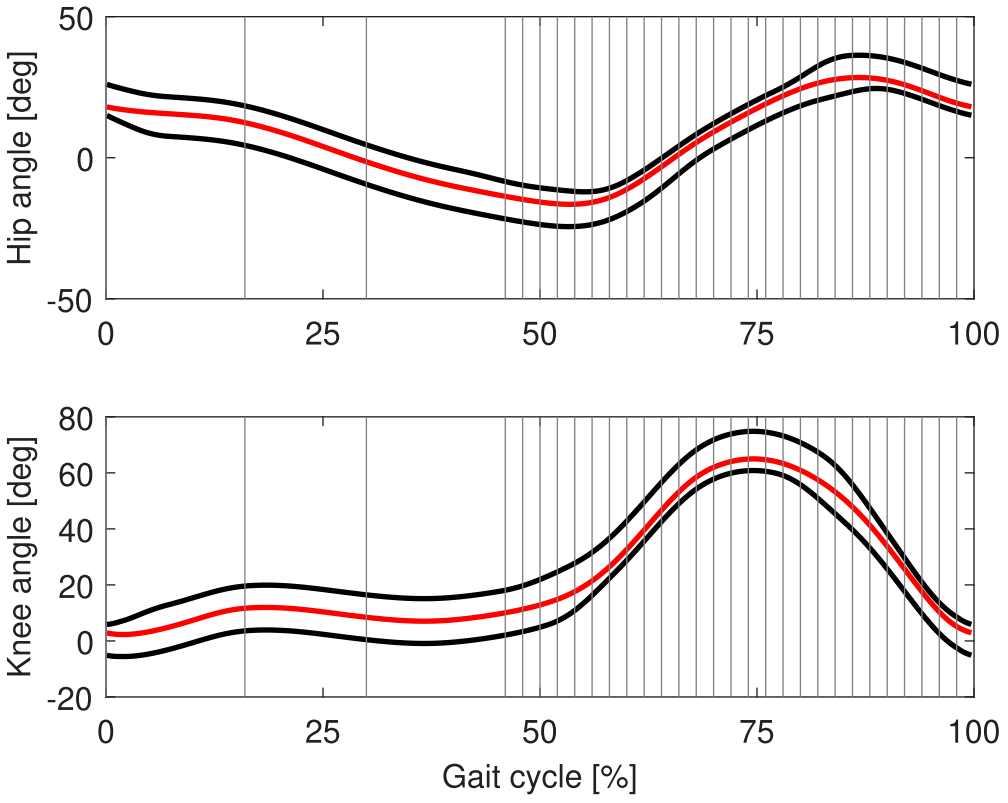 |
| --- |
| B  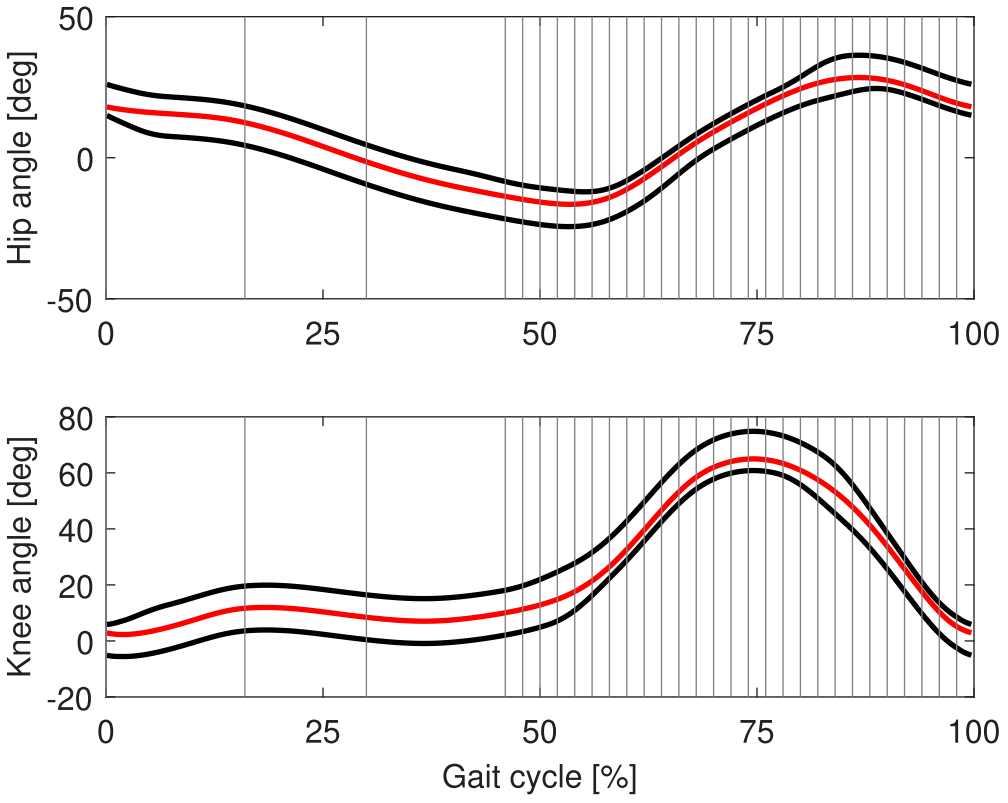 |

Figure A.3.1: In the AAN controller hip and knee thresholds are defined as shown in the graph A (hip angle) and B (knee angle). The reference trajectory (red) is taken from [1], but it can be adjusted from the Lokomat user interface (see Figure A.3.2.). The thresholds (black lines) are defined ad-hoc to allow variability in non-critical phases of the gait cycle and ensure safety where the deviations need to be minimal. The thresholds width ranges from 9° to 14° for the hip angular trajectory and from 11° to 18° for the knee angular trajectory. Thresholds are the same among all subjects. The gait cycle is divided in 30 windows (grey lines show the windows’ limits).

The reference trajectories for hip and knee were adjusted for every patient during the familiarization session and kept fixed in the following session. The Lokomat therapist interface allows limited adjustments of the predefined gait pattern: range of motion (ROM) and offset of the hip and knee joint can be manually tuned in a discrete manner within certain limits. Adapting the ROM has a specific effect on the maximum joint angle. Adapting the offset shifts the entire joint trajectory towards flexion or towards extension.

These settings allow to personalize to a certain extent the trajectory for each patient (e.g. some patients walk with a longer step length, that can be achieved by increasing the hip range of motion). During the familiarization session, as it is common practice in clinics, we tried to determine the reference trajectory of the Lokomat so that it was as close as possible to the patient’s physiological trajectory. We used the AAN display as guidance (Figure 3 of the paper): here reference and actual trajectory are shown. We adapted the Lokomat reference trajectory in order to match the reference to the actual trajectory displayed on the screen. Ideally, reference and actual trajectory should overlap. The adapted reference trajectories for hip and knee used during the experiment are displayed in Figure A.3.2.

| A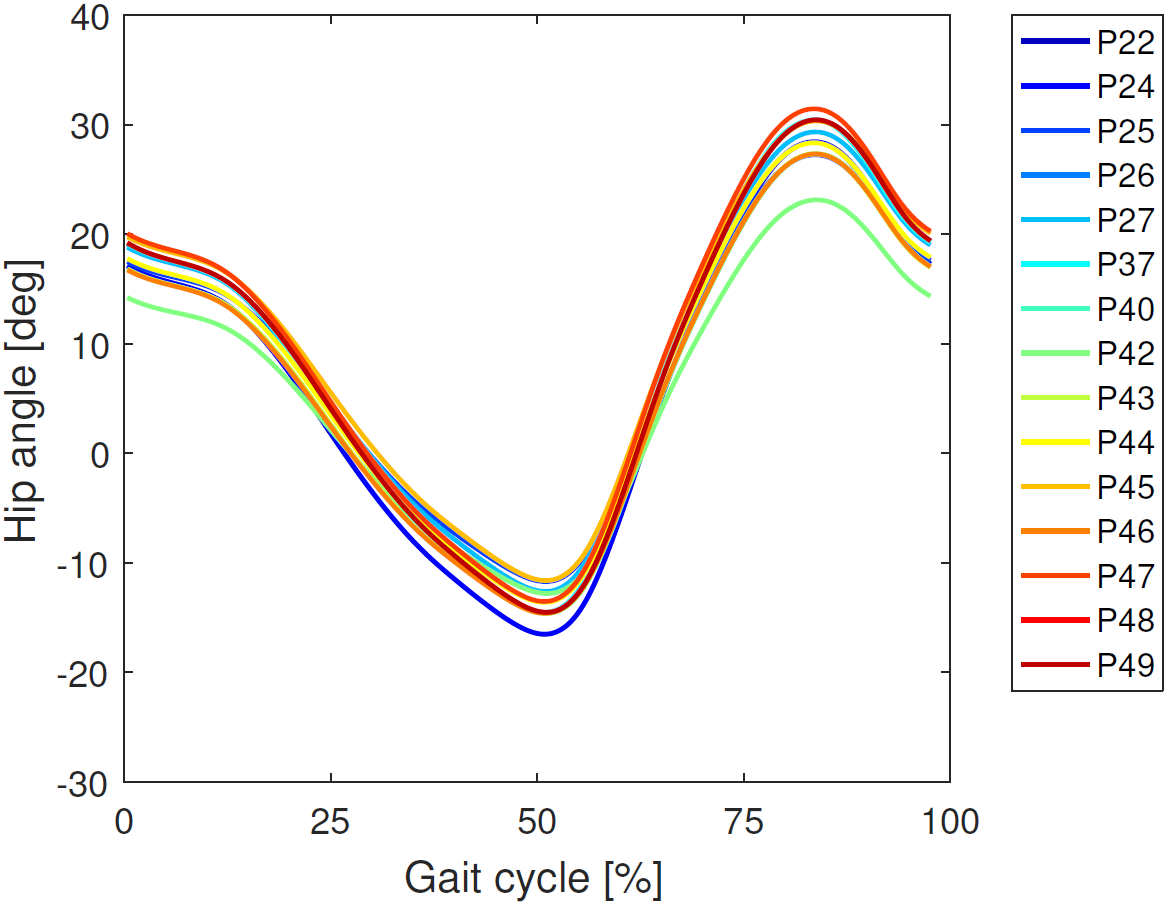 | B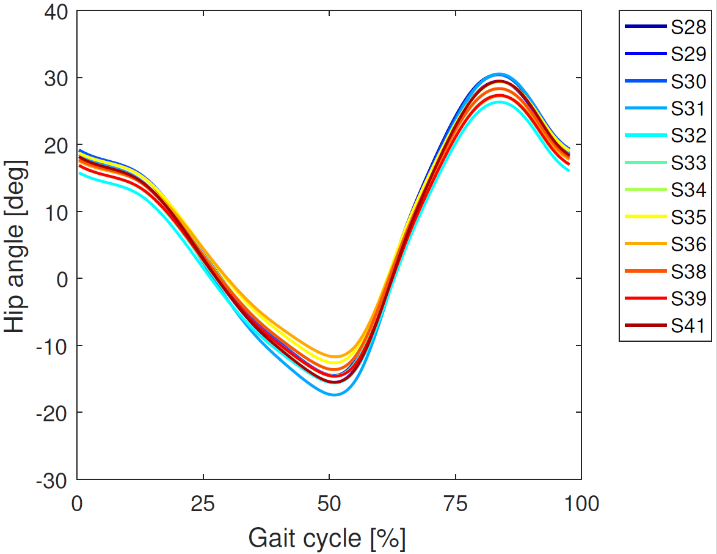 |
| --- | --- |
| C  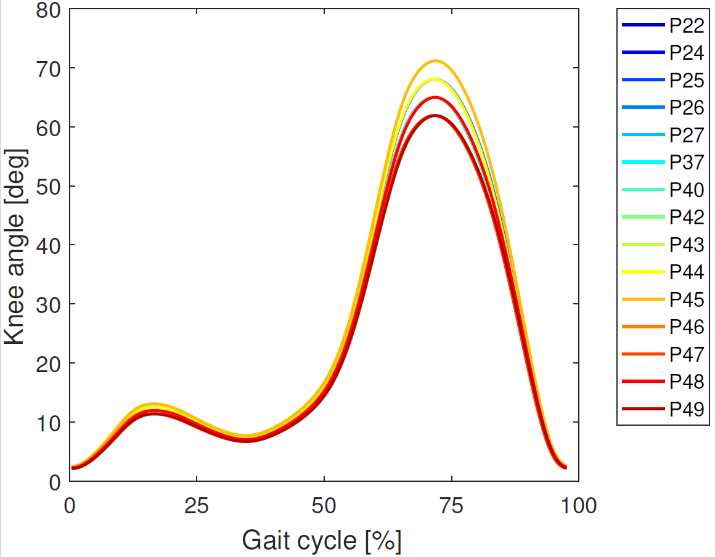 | D  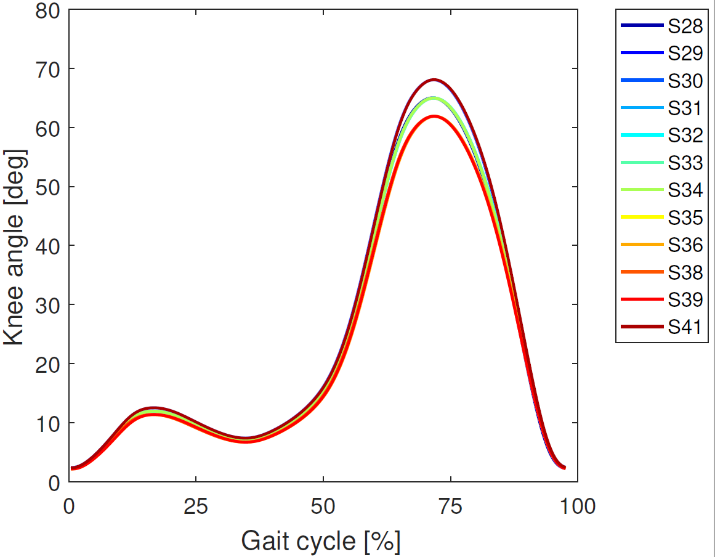 |

Figure A.3.2: Hip (A and B) and knee (C and D) reference trajectories determined for each patient (A and C) and able-bodied subject (B and D) during the familiarization session. The minimum hip ROM applied was 36°, while the maximum 48°. The minimum knee ROM applied was 60°, while the maximum 69°.

**References**

1. Colombo, G., et al., *Treadmill Training of Paraplegic Patients Using a Robotic Orthosis.* J Rehabil Res Dev, 2000. **37**(6): p. 693-700.
